# Support Strategies to Enhance Adherence to a Prescription Digital Therapeutic for Erectile Dysfunction: Retrospective Quasi-Experimental Cohort Study

**DOI:** 10.2196/76724

**Published:** 2026-07-14

**Authors:** Leo Dieter, Mara Haschke, Kurt Miller, Laura Elisa Wiemer

**Affiliations:** 1 Department of Urology Charité - Universitätsmedizin Berlin Berlin Germany; 2 Technical University of Munich Munich Germany

**Keywords:** adherence, behavioral therapy, DiGA, digital health, digital therapy, Digitale Gesundheitsanwendung, erectile dysfunction, mHealth, mobile health, nudging, SMS, telephone intervention

## Abstract

**Background:**

Sustained engagement is a central challenge for digital therapeutics. In routine operations, inactivity-triggered supports (eg, SMS reminders and telephone follow-up) are used to nudge patients back after lapses, but evidence in digital erectile dysfunction (ED) therapy is limited.

**Objective:**

This study aims to evaluate whether low-threshold support in the form of SMS reminders and structured telephone outreach, both triggered after 7 or more days of inactivity, improves engagement and patient-centered outcomes in a certified German digital health application (Digitale Gesundheitsanwendung [DiGA]) for ED.

**Methods:**

In a pragmatic, calendar-time (rotating-week) quasi-experimental allocation, 470 men with physician-diagnosed ED entered 1 of 3 groups: control (no additional support), SMS (automated reminders), or call (structured telephone contact). Analyses were conducted as assigned. The primary end point was active weeks (number of weeks with ≥1 completed session) during the 12-week study period, derived from app logs with built-in completion rules. A prespecified mechanistic end point captured any reactivation after an inactive week (yes or no). Secondary end points included Clinical Global Impression-Improvement (CGI-I), week-12 5-item International Index of Erectile Function (IIEF-5), intention to continue therapy, and documented continuation (“conversion”) within 3 months. Covariate-adjusted models included age, BMI, smoking, and baseline pharmacotherapy; calendar-week sensitivity was planned.

**Results:**

Participants completed a mean of 6.34 (SD 4.44) active weeks. Unadjusted means favored both support groups (control: 5.79; call: 6.57; SMS: 6.80); the prespecified SMS-vs-control contrast was nominally significant (*P*=.049, small effect). In covariate-adjusted models, planned pairwise contrasts were not significant. In a calendar-adjusted sensitivity model, the omnibus group term reached significance, but adjusted pairwise contrasts remained nonsignificant; stricter adherence definitions led to the same overall inference. By contrast, any reactivation after inactivity was more common in the intervention groups than in the control group (omnibus chi-square test *P*=.008), consistent with the intended mechanism of breaking inactivity spells. The call group showed a higher intention to continue therapy (49% vs 38% in the control arm; *P*=.04); conversion did not differ. CGI-I and IIEF-5 showed no arm-wise differences over 12 weeks; follow-up availability was limited in routine care.

**Conclusions:**

Inactivity-triggered supports in a real-world ED DiGA reactivated use after lapses, and telephone outreach increased motivation to continue, while effects on weekly dose were small and not significant after adjustment, and clinical outcomes did not differ over 12 weeks. Programs may consider a stepped approach (SMS first-line nudge and call as escalation) and target system bottlenecks that decouple motivation from realized continuation. Further work should test longer-term outcomes, targeting, and generalizability.

## Introduction

The increasing digitization of the health care system, particularly through the Digital Healthcare Act (Digitale-Versorgung-Gesetz [DVG]), has allowed physicians in Germany to prescribe digital health applications (Digitale Gesundheitsanwendung [DiGA]) since December 2019. These applications offer innovative approaches to improving patient care and are specifically designed to close outpatient care gaps and increase treatment quality.

A crucial factor for the success of digital therapy approaches is therapy adherence, defined as the extent to which patients consistently and sustainably follow medical and therapeutic recommendations [1]. In the case of erectile dysfunction (ED), a condition involving both physical and psychological components, continuous engagement with the therapeutic content is essential for successful treatment outcomes [2,3]. In practice, however, many patients discontinue digital therapies prematurely, often due to a lack of motivation, insufficient personal support, or technical barriers [4].

To address this challenge, nudging strategies, such as automated SMS reminders or personal telephone calls, are gaining increasing attention. Studies have shown their effectiveness in enhancing adherence in chronic conditions [5-8]. Nevertheless, there is still a lack of studies specifically investigating the benefits of such interventions in the context of digital ED therapy.

The DiGA Kranus Edera, which has been permanently listed in the DiGA directory since 2023 [9] based on its randomized controlled trial results, follows a holistic treatment approach that combines physical exercises with psychological modules in a mobile app [10,11]. Building on this foundation, the present retrospective evaluation investigates whether digital support measures, such as SMS reminders or telephone calls, can improve adherence and motivation during the course of this digital therapy.

This study aims to analyze the effect of such strategies on therapy adherence (primary end point: active training weeks) as well as on other patient-related outcomes (eg, Clinical Global Impression-Improvement [CGI-I], continuation intent, and actual therapy continuation). Furthermore, the study investigates whether certain sociodemographic or clinical factors, such as age, BMI, or preexisting conditions, affect adherence. The findings aim to contribute to the optimization of digital therapy concepts and more personalized patient engagement in the digital health care setting [12].

## Methods

### Ethical Considerations

This retrospective real-world analysis of the Kranus Edera digital therapy program was reviewed by the Charité - Universitätsmedizin Berlin Ethics Committee (Ethikausschuss am Campus Benjamin Franklin) and approved under protocol EA4/104/24 (committee vote: December 9, 2024; confirmation letter: December 16, 2024). The study analyzed secondary, deidentified usage data exported from routine operations; no research procedures were conducted by the academic team. At program enrollment, users agreed to the provider’s terms and privacy policy permitting the use of deidentified data for quality improvement and research. The ethics committee confirmed that the original consent covers this secondary analysis; therefore, no additional consent was required. Participants could opt out of SMS or telephone contact during routine operations; such preferences were honored by the provider. Before transfer to the academic team, all records were deidentified (ie, no names, telephone numbers, or other direct identifiers). Operational messaging and call logs containing identifiers were not shared. Analyses were conducted under a data use arrangement compliant with the General Data Protection Regulation (GDPR) and institutional policies; only deidentified aggregate data are reported. No compensation was provided for this retrospective secondary analysis; participants received standard program communications (SMS messages and calls) as part of routine care.

### Study Design

This evaluation is a quasi-experimental retrospective cohort analysis based on anonymized usage data from the Kranus Edera DiGA, exported from the provider’s internal database. Data from patients who began therapy between May 28, 2023, and October 20, 2023, were included; the observation window covered 12 weeks from therapy initiation. The support strategies (telephone calls and SMS reminders) were implemented by the provider as part of routine operations; the academic team did not design or assign these interventions and analyzed their real-world use post hoc. The aim was to compare adherence and clinical outcomes among the call, SMS, and control (no additional support) groups. The study protocol was approved by the Ethics Committee of Charité - Universitätsmedizin Berlin (EA4/104/24; Multimedia Appendix 1). All data were fully anonymized prior to analysis and processed in accordance with the Declaration of Helsinki and the GDPR.

### Hypotheses

Based on existing literature on digital health interventions and adherence behavior, the following hypotheses were prespecified for this study:

- Patients receiving support interventions (telephone calls or SMS reminders) will demonstrate significantly higher therapy adherence, operationalized as the number of active weeks during the 12-week program, compared with patients in the control group without support.
- Patients in the support groups will report higher subjective treatment satisfaction, measured using the CGI-I scale, and greater improvement in erectile function, measured by the change in 5-item International Index of Erectile Function (IIEF-5) scores, compared with patients in the control group.
- Patients receiving support interventions will show a higher intention to continue the therapy after the initial 12-week program compared with patients without support.
- Higher therapy adherence (number of active weeks) will be positively associated with subjective treatment improvement (CGI-I) and real-world therapy continuation behavior (conversion rate).

### Group Assignment

Participants were allocated at enrollment by the provider’s prespecified weekly rotation used in routine operations. The unit of allocation was the calendar week (no individual randomization by the research team):

- Week W: telephone support
- Week W+1: SMS support
- Week W+2: usual care or control (no proactive outreach)
- Week W+3: telephone support, and the sequence repeats

Assignment depended only on each participant’s enrollment date (therapy initiation) and was independent of individual characteristics. This pragmatic scheme minimized operational burden and contamination between groups while creating quasi-experimental variation. Because assignment was tied to calendar time, primary analyses adjusted for prespecified baseline covariates and were complemented by calendar-adjusted sensitivity models that included a standardized enrollment-time covariate. We also examined robustness to calendar time using alternative time codings and exploratory interaction tests. All analyses were conducted as assigned (intention-to-treat).

### Clinical Eligibility

To align the cohort with the product’s intended population in routine care, we included:

- Male adults (aged ≥18 years) with physician-confirmed ED
- Baseline IIEF-5 at therapy initiation with an IIEF-5 score of ≤21 (at least mild ED)
- First-time Kranus Edera use within the study window (May 28, 2023-October 20, 2023)

Definition and rationale: the IIEF-5 is a validated questionnaire assessing erectile function (total score: 5-25, higher scores indicate better erectile function; 4-week recall period). A cutoff score of ≤21 corresponds to at least mild ED (no ED, 22-25; mild, 17-21; mild-to-moderate, 12-16; moderate, 8-11; severe, 5-7) and is commonly used to identify clinically relevant ED.

### Analytic Exclusions

From the eligible pool, we excluded:

- Repeat users within the study window
- Records lacking the minimum variables required for analysis (eg, all weekly usage data missing)

Comorbidities (eg, hypertension and diabetes) were not exclusion criteria; they were described and, where appropriate, used as covariates.

### Prescribing Context and Contraindications

Kranus Edera is prescribed in Germany through the DiGA pathway. Prescribers are responsible for indication and contraindication screening according to the product’s instructions for use. Because this was a retrospective, deidentified data export, individual prescription records were not available; eligibility was operationalized using the variables above. A full list of contraindications and precautions (eg, unstable angina, recent myocardial infarction, New York Heart Association class III-IV heart failure, high-risk arrhythmias, etc, with *ICD-10* [*International Statistical Classification of Diseases, Tenth* Revision] codes where applicable) is provided in Multimedia Appendix 2.

### App Structure and Modules

#### Overview

Kranus Edera delivers a 12-week, guided digital therapy for ED, combining (1) basic pelvic floor muscle training (PFMT), (2) pelvic floor physiotherapy, (3) cardiovascular endurance sessions, (4) brief mindfulness and mental exercises (eg, breathing and stress regulation), and (5) educational and sexual therapy content (knowledge modules on ED etiology, lifestyle, medication, and partner communication). The program is progressive: difficulty and training load increase across weeks, and the app adapts suggested tasks based on completion and self-reported ease.

- Pelvic floor (PFMT): short, technique-focused basic drills (approximately 3-7 minutes) emphasizing anterior pelvic floor activation, relaxation, and coordination.
- Cardiovascular training: time-based walking or aerobic sessions with targets expressed in seconds or minutes (approximately 10-30 minutes), aligned with guideline-consistent aerobic activity to support endothelial function.
- Physiotherapy: longer guided physiotherapy videos (approximately 5-10 minutes) to facilitate PFMT and physical activity.
- Mental exercises: brief (approximately 5 minutes) audio-guided breathing and mindfulness exercises to reduce performance anxiety and support adherence.
- Knowledge and education: lessons of approximately 3-7 minutes on ED mechanisms, risk factors, medication classes, lifestyle, and communication strategies.

#### Session Cadence and Examples

Typical weekly plans include multiple short sessions (eg, 3-5 PFMT or physiotherapy tasks, 1-3 cardiovascular sessions totaling approximately 20-90 minutes across the week, 1-2 mental exercises, and 1-7 knowledge modules). Example exercises include supine pelvic contractions with breath cueing, bridges, hip openers, interval running cardio, 4-7-8 breathing, and myth-busting lessons on ED. A week-by-week module map with example tasks and nominal durations is provided in Multimedia Appendix 3.

#### Standards and Prior Evidence

The app’s multidomain design (PFMT, aerobic activity, education, and psychological support) mirrors guideline recommendations for multimodal ED management and demonstrated clinically meaningful improvements in IIEF-5 in a randomized, single-blind controlled trial of this app (EDDIG) [10].

### Description of the Support Interventions

We evaluated 2 inactivity-triggered, weekly reactivation strategies implemented in routine operations.

Telephone support (call arm): when a participant had ≥7 consecutive inactive days (no logged activity in any domain), an automated flag prompted outreach. Calls were delivered by technical support staff from the provider’s digital therapeutics support team (nonclinical), trained in basic troubleshooting and motivational interviewing and working according to a standardized standard operating procedure. Trained staff attempted up to 3 calls per episode (same day, next day, and the following day), between 9 AM and 7 PM local time. Calls followed a standardized opener and brief problem-solving script (technical troubleshooting or motivational and organizational support), aiming to set 1 concrete activity within 48 hours; red-flag symptoms prompted escalation. Participants could opt out of telephone contact. In routine operations, outreach cycles were repeated if inactivity persisted and were typically capped after 3 consecutive weeks of nonengagement.

SMS support (SMS arm): a weekly automated check sent 1 prescripted, week-specific SMS message when ≥7 inactive days were present. Messages were delivered in the morning (7 AM-11 AM), normalized short lapses, and prompted 1 short, week-appropriate module. The channel was one-way by design; replies were handled ad hoc during routine operations. Participants could opt out of SMS messages. As in the call arm, rechecks occurred weekly, and SMS outreach was typically capped after 3 consecutive weeks of persistent inactivity.

Operational rationale for the 7-day trigger: the ≥7-day threshold was defined by the provider for standard operating procedure simplicity and operational feasibility (weekly check and weekly nudge) and to target early reactivation after a full week of nonuse. This retrospective study did not set that threshold; we analyzed its real-world use. Notably, the same 7-day lapse key performance indicator has been used in prior digital adherence work (eg, medication refill gaps and SMS nudging frameworks) and in broader digital health reengagement strategies [6-8].

Design intent and end point alignment: both support strategies were designed to reactivate participants once per week rather than escalate intensity daily. Accordingly, the primary adherence end point was active weeks (≥1 activity per week), and the mechanism-aligned end point was reactivation after prior inactivity (active in week t given inactivity in week t–1). Stricter intensity and dose metrics are reported as sensitivity or exploratory outcomes.

### Measurement of the Primary End Point

#### Overview

The prespecified primary end point was the number of active weeks (range 0-12 weeks) during the 12-week program. A week was considered active if the user completed ≥1 training session; multiple sessions in the same week still counted as 1 active week. Session completion required passing app-enforced criteria (completion of the full exercise flow and confirmation screens); merely opening the app or brief taps did not qualify. Participants remained in their assigned group according to the intention-to-treat principle; weeks with no telemetry were counted as inactive.

#### Link to the Intervention’s Reactivation Mechanism

Outreach (telephone or SMS) was triggered only after ≥7 consecutive inactive days, with the goal of weekly reactivation. Completion of any session paused further outreach until the next inactivity episode. Thus, active weeks directly capture the intended proximal effect (breaking inactivity within the program’s weekly cadence).

This operationalization is consistent with established practice in digital health evaluations that derive adherence from objective usage traces rather than self-report [13] and aligns with payer reporting in Germany, where weekly engagement is a core indicator in DiGA evaluations (eg, Techniker Krankenkasse annual reports [14]).

#### Robustness Checks

We additionally report (1) the reactivation rate, defined as the proportion of weeks t that were active given that week t–1 was inactive (denominator = all person-weeks following an inactive week); (2) active-weeks version 2 (≥2 sessions or ≥30 minutes of cardiovascular exercise); (3) multidomain engagement (≥2 of 5 weekly goals met); and continuous dose measures (total sessions and median sessions per active week). These thresholds reflect the program’s recommended cadence (3-5 physiotherapy or PFMT sessions, 1-3 cardiovascular sessions, and mental exercises and education). Adherence derivation steps (pseudocode) are provided in Multimedia Appendix 3.

### Measurement of Secondary End Points

#### CGI-I

To evaluate patients’ subjective impression of change, a modified version of the CGI-I scale was used. At the end of the 12-week program, participants were asked via an email-based questionnaire, “How would you rate your current erectile function compared to before the therapy?”

Responses were given on a 7-point scale from 1=very much worse to 7=very much improved. For consistency with the original CGI-I interpretation, scores were recoded so that lower values indicated greater improvement. The scale was used in a self-rated, ED-specific format, in line with prior validation studies [15,16].

#### IIEF-5 (Erectile Function) and ΔIIEF-5

Erectile function was measured with the IIEF-5, a validated patient-reported outcome assessing erectile function over the past 4 weeks (items: erection confidence, firmness, penetration ability, maintenance, and intercourse satisfaction) [17]. Each item is scored from 1 to 5; the total score ranges from 5 to 25, with higher scores indicating better erectile function. Common severity bands were as follows: no ED, 22-25; mild, 17-21; mild-to-moderate, 12-16; moderate, 8-11; and severe, 5-7. Baseline IIEF-5 was collected at therapy initiation; a follow-up IIEF-5 was offered voluntarily in the app during week 12. Change in erectile function (ΔIIEF-5) was computed as the week-12 score minus the baseline score, so positive values reflected improvement. Baseline IIEF-5 was also used as a covariate in adjusted outcome models.

#### Intention to Continue Therapy

Patients’ intention to continue the digital intervention was assessed at the end of the 12-week program via a standardized email-based survey. Participants were asked the following dichotomous question: “Would you like to continue the therapy?” (response options: “yes” or “no”).

Although this single-item question is not part of a validated instrument, it serves as a pragmatic proxy for patient satisfaction and perceived utility of the intervention. In line with previous adherence research, stated intention has been interpreted as an early indicator of behavioral engagement and treatment acceptance, particularly in digital health contexts where user motivation is critical to sustained use [12]. The variable was coded as binary (1=yes and 0=no).

#### Therapy Continuation Rate

Actual therapy continuation, also referred to as conversion, was operationalized based on patients’ real-world therapy behavior. Using the manufacturer’s internal database, it was determined whether a participant continued the same therapy within 3 months after completion of the initial 12-week therapy cycle.

This binary outcome (1=therapy continued and 0=no continuation) reflects objective, externally validated treatment behavior beyond self-report. As a behavioral end point, it offers insight into long-term therapeutic adherence and real-world uptake of the intervention under routine care conditions.

### Statistical Analysis

#### Overview

All analyses followed an intention-to-treat principle based on calendar-week operational allocation (control, call, and SMS). Because assignment followed calendar-time operational changes, we adjusted for baseline covariates (age, BMI, smoking status, and baseline IIEF-5 where applicable) and acknowledge the possibility of residual time-trend confounding. We prespecified a calendar-time sensitivity analysis in which the primary adherence model additionally included a standardized enrollment-time covariate; we report the omnibus group test and Bonferroni-adjusted pairwise contrasts from that model. Exploratory analyses assessed alternative time codings and group-by-time interactions.

#### Descriptive Statistics

We summarized the total sample and the groups (control, call, and SMS) with means and SDs. Distributional diagnostics (skewness and kurtosis, histograms, and probability-probability plots) informed model choice. With n>30 per group, 2-tailed *t* tests and general linear models (GLM) were considered robust to moderate nonnormality; BMI showed slight positive skewness but remained within acceptable bounds.

#### Primary Adherence End Point (Mechanism-Aligned)

The prespecified primary end point was active weeks (number of weeks with ≥1 completed session, 0-12). Group differences were tested with independent-samples *t* tests (call vs control; SMS vs control) and one-way GLM or analysis of covariance (ANCOVA) with group as the factor and age, BMI, and smoking status as covariates. We report adjusted means (estimated marginal means), 95% CIs, and Bonferroni-adjusted pairwise comparisons.

#### Clinical Outcomes (IIEF-5 and CGI-I)

The primary model for IIEF-5 was an ANCOVA of week-12 IIEF-5 with baseline IIEF-5 (and age, BMI, and smoking status) as covariates. This approach is preferred to testing change scores because it typically offers greater precision when baseline and follow-up are correlated and helps mitigate baseline imbalance and regression to the mean in nonrandomized cohorts.

- Sensitivity: we additionally report ΔIIEF-5 = week 12 − baseline (*t* tests or GLM) for comparability with prior work; interpretation prioritizes the ANCOVA results.
- CGI-I: analyzed directly using a GLM or ANCOVA with the same covariates (CGI-I is itself an improvement scale).

For the ANCOVA models, we checked homogeneity of regression slopes, residual normality, and influential observations; conclusions were unchanged.

#### Intent to Continue and Conversion

Group differences in intent to continue (implementation outcome) were tested using chi-square tests and logistic regression with group as the main factor and age, BMI, and smoking status as covariates (reporting adjusted odds ratios [ORs] and 95% CIs). Conversion (documented continuation) was analyzed analogously. Because responses were voluntary, these were treated as complete-case outcomes, and response rates are reported; we do not infer clinical efficacy from intention.

#### Additional Adherence Measures

Threshold-based adherence was assessed as the proportion of participants achieving ≥6, ≥8, and ≥9 active weeks using chi-square tests, with adjusted risk differences and ORs reported with 95% CIs. Adherence intensity was evaluated as the median number of sessions per active week using nonparametric tests alongside GLMs when the data were skewed. Domain-specific goal attainment was assessed as the number of weeks participants met weekly goals in pelvic floor training, pelvic floor physiotherapy, cardiovascular exercise, mental exercises, and knowledge modules, as well as the percentage of all weekly goals achieved across the 12-week intervention, using GLMs or chi-square tests as appropriate. Additional adherence metrics included the number of “stricter” active weeks (version 2), defined as ≥2 sessions per week or ≥30 minutes of cardiovascular exercise per week, and the number of multidomain weeks, defined as weeks in which participants met goals in at least 2 of the 5 intervention domains.

#### Mechanism-Specific Reactivation and Persistence

Reactivation was defined as being active in week t given inactivity in week t−1. Among participants with ≥1 opportunity, we compared any reactivation (yes or no) using chi-square tests and modeled the reactivation rate (reactivations and opportunities) with ANCOVA using the same covariates.

Persistence, defined as time to the first inactive week, was analyzed using Kaplan-Meier analysis with log-rank tests; participants who remained active throughout the 12 weeks were censored at week 12. We also computed the longest active streak.

#### Missingness, Nonresponder, and Bias Checks

Primary models used complete cases for the end point of interest; descriptive analyses used pairwise deletion. We compared baseline characteristics and adherence between responders and nonresponders (follow-up completion) using *t* tests and chi-square tests and fitted a logistic regression predicting follow-up completion (covariates plus group) to assess differential missingness by group.

#### Sensitivity Analyses

We repeated key analyses excluding participants reporting baseline pharmacotherapy (any phosphodiesterase type 5 [PDE-5] inhibitors, Schwellkörper-Autoinjektionstherapie [SKAT], or medicated urethral system for erection [MUSE]). When assumptions were questionable, we added nonparametric companion analyses (Mann-Whitney or Kruskal-Wallis).

All tests were 2-sided with α=.05. We report effect sizes (mean differences, adjusted mean differences, ORs, and hazard ratios where applicable) with 95% CIs. Analyses were conducted using SPSS (version 30; IBM Corp) with the GLM, univariate ANOVA, Kaplan-Meier survival, and logistic regression procedures.

## Results

### Description of the Study Population

The study population consisted of 470 men with physician-diagnosed ED. The mean baseline IIEF score was 13.1 (SD 4.65), indicating moderate to severe ED. Thus, this cohort represents a clinically relevant population with typical ED characteristics.

The average BMI was 27.3 (SD 4.33) kg/m^2^, which falls within the preobesity range according to the World Health Organization (WHO) classification [18]. This value is comparable to the average BMI of German men aged 45-64 years (approximately 27.5-27.7 kg/m^2^) [19].

Participants were, on average, aged 48 (SD 14.2) years, with a broad age distribution centered in middle adulthood, an age group in which ED is increasingly common and often overlaps with metabolic risk factors [20].

Functional capacity was high, with 83% (390/470) of participants able to perform 40 minutes of cardiovascular exercise and 96% (451/470) able to move independently.

This high level of functional independence suggests that, despite comorbidities, the cohort was predominantly physically active.

These prevalences align with known risk factors for ED and are partially elevated compared with the general German male population, consistent with the clinical nature of this sample [21]. Nearly half of the men used PDE-5 inhibitors; other invasive or pharmacological therapies, such as SKAT or MUSE, were rare.

### Baseline Group Comparability

Baseline demographic and clinical characteristics were broadly similar across groups (usual care [control], telephone support, and SMS). Continuous variables are summarized as mean (SD) and compared descriptively; where hypothesis tests are reported, one-way ANOVA was used for continuous variables and chi-square tests (or Fisher exact tests where expected cell counts were sparse) for categorical variables. To quantify balance independent of sample size, we report absolute standardized mean differences alongside *P* values.

The mean age was 48 (SD 14.2) years, and the mean BMI was 27.3 (SD 4.3) kg/m^2^, with no between-group differences (ANOVA *P*=.74 and *P*=.89, respectively). Functional capacity and independence were high across groups (able to perform 40 minutes of cardiovascular exercise: 390/470, 83%; independent mobility: 451/470, 96%). The prevalence of common comorbidities was comparable, including hypertension (129/470, 27.4%), dyslipidemia (93/470, 19.8%), and diabetes mellitus (51/470, 10.9%). Baseline pharmacotherapy also did not differ significantly between groups (PDE-5 inhibitor use: 230/470, 48.9% overall; SKAT or MUSE: 6/470, 1.3%; any pharmacotherapy approximately 50%; all chi-square tests: not significant). Key factors relevant to outcomes and missingness (current smoking: 111/470, 23.6%; history of prostatectomy: 34/470, 7.2%) were similarly distributed.

Between-group differences at baseline were small by ANOVA or chi-square testing. For the prespecified covariates (age, BMI, current smoking, baseline pharmacotherapy, and baseline IIEF-5), no statistically significant differences were detected (Table 1). Primary models adjusted for age, BMI, smoking status, and baseline pharmacotherapy.

**Table 1:** Baseline characteristics by group. Values are mean (SD) or n (%). *P* values are from one-way ANOVA (continuous variables) or chi-square tests (categorical variables). Denominators vary because of item-level missingness; percentages are based on the nonmissing denominator for each variable.

Characteristic	Total (N=470)	Control (n=180)	Call (n=150)	SMS (n=140)	*P* value
Age (years), mean (SD)	48 (14.2)	48.3 (14.5)	48.4 (13.6)	47.2 (14.6)	.74
BMI (kg/m^2^), mean (SD)	27.3 (4.3)	27.4 (4.4)	27.4 (4.6)	27.2 (4.0)	.89
Able to do 40 minutes of cardiovascular exercise, n (%)	390 (83)	144 (80)	123 (82)	123 (87.9)	.17
Able to move independently, n (%)	451 (96)	171 (95)	144 (96)	136 (97.1)	.63
History of spinal injury, n (%)	28 (6)	12 (6.7)	9 (6)	7 (5)	.82
Circulatory disorder, n (%)	14 (3)	9 (5)	2 (1.3)	3 (2.1)	.12
Diabetes, n (%)	51 (10.9)	21 (11.7)	19 (12.7)	11 (7.9)	.38
Dizziness, n (%)	33 (7)	11 (6.1)	13 (8.7)	9 (6.4)	.63
Elevated blood lipids, n (%)	93 (19.8)	45 (25)	26 (17.3)	22 (15.7)	.08
Hypertension, n (%)	129 (27.4)	48 (26.7)	40 (26.7)	41 (29.3)	.84
Pulmonary issues, n (%)	47 (10)	16 (8.9)	17 (11.3)	14 (10)	.76
Mental health burden, n (%)	112 (23.8)	42 (23.3)	38 (25.3)	32 (22.9)	.87
History of spinal surgery, n (%)	52 (11.1)	18 (10)	21 (14)	13 (9.3)	.37
History of prostatectomy, n (%)	34 (7.2)	14 (7.8)	11 (7.3)	9 (6.4)	.90
Knee instability, n (%)	121 (25.7)	55 (30.6)	38 (25.3)	28 (20)	.01
Smoker, n (%)	111 (23.6)	46 (25.6)	38 (25.3)	27 (19.3)	.35
Takes PDE-5^a^ inhibitors, n (%)	230 (48.9)	93 (51.7)	67 (44.7)	70 (50)	.43
Uses SKAT^b^ or MUSE^c^, n (%)	6 (1.3)	2 (1.1)	3 (2)	1 (0.7)	.60

^a^PDE-5: phosphodiesterase type 5.

^b^SKAT: Schwellkörper-Autoinjektionstherapie.

^c^MUSE: medicated urethral system for erection.

### Primary Outcome: Therapy Adherence

#### Definition and Hypothesis

Therapy adherence was prespecified as the number of active weeks (range 0-12 weeks) during the 12-week program; a week was “active” if the user completed ≥1 training session in the app (completion enforced by app logic). The confirmatory hypothesis specified 2 planned contrasts, call vs control and SMS vs control, with α=.05 (2-sided) for each contrast.

#### Descriptives

**Figure 1:**
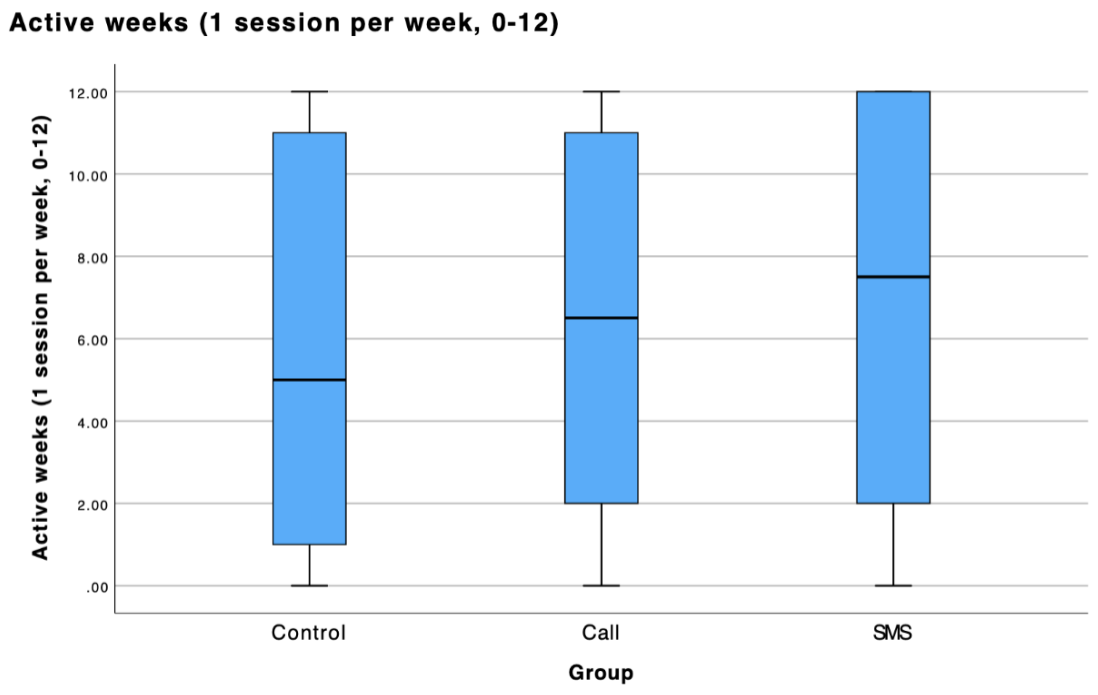
Boxplot of active training weeks by group. The plot displays the distribution of weekly exercise adherence across the control, call, and SMS groups. Median adherence was highest in the SMS group, with slightly lower variability than in the control group.

Across the cohort (N=470), participants completed a mean of 6.34 (SD 4.44) active weeks. Group distributions were approximately symmetric, with small skewness in each group. The median number of active weeks was 5.0 weeks in the control group, 6.5 weeks in the call group, and 7.5 weeks in the SMS group. Figure 1 shows the unadjusted distribution of active weeks by group.

#### Unadjusted Planned Contrasts

Group means were 5.79 (SD 4.54) for the control group, 6.57 (SD 4.22) for the call group, and 6.80 (SD 4.48) for the SMS group. The omnibus one-way ANOVA suggested a trend (*F*_2,467_=2.333; *P*=.09). Prespecified pairwise tests vs the control group were as follows: call vs control, Δ=0.78 weeks (95% CI −0.17 to 1.73; *P*=.11, not significant); SMS vs control, Δ=1.01 weeks (95% CI 0.01-2.00; *P*=.049); Cohen *d* for SMS vs control was 0.22 (small). Bonferroni-adjusted GLM pairwise comparisons were nonsignificant.

#### Adjusted analyses

GLM models adjusted for age, BMI, smoking status, and baseline pharmacotherapy showed no significant planned pairwise contrasts in active weeks; the unadjusted SMS advantage was attenuated after adjustment. In a calendar-adjusted sensitivity that additionally included a standardized enrollment-time covariate, the omnibus group effect was small but statistically significant, whereas Bonferroni-adjusted pairwise contrasts remained nonsignificant. Corresponding adjusted estimates are reported in Multimedia Appendix 4.

#### Sensitivity Analyses

Using stricter adherence definitions (≥2 sessions per week or ≥30 minutes of cardiovascular exercise per week), a multidomain engagement definition (≥2 of 5 weekly goals), and intensity measures (total sessions and median sessions per active week) yielded the same conclusion, with no significant adjusted group differences.

#### Mechanism-Aligned Proximal Signal

Unadjusted, the call and SMS groups showed higher mean reactivation rates than the control group (control group 0.167; call group 0.223; SMS group 0.235). In a GLM adjusted for age, BMI, smoking status, and baseline pharmacotherapy, between-group differences in the reactivation rate were small and not significant (*F*_2,467_=1.438; *P*=.24; partial η^2^=0.008). By contrast, the probability of any reactivation differed across groups on an unadjusted 3 × 2 test (*χ*^2^_2_=9.7; *P*=.008), with higher proportions in call and SMS groups than in the control group.

#### Persistence and Clinical Outcomes

Time to first inactivity did not differ between groups (log-rank *P*=.36). In ANCOVA of week-12 IIEF-5 adjusted for baseline IIEF-5, age, BMI, smoking status, and baseline pharmacotherapy, the group effect was not significant; adjusted pairwise comparisons were also nonsignificant. Table 2 summarizes the unadjusted group means for the primary and secondary outcomes for transparency; inferential conclusions rely on the adjusted models described above.

**Table 2:** Primary and secondary outcomes by group (N=470).

Outcome	Total (N=470)	Control group	Call group	SMS group	*P* value (omnibus across groups)	*P* value (control vs call)	*P* value (control vs SMS)
Active training weeks (adherence), mean (SD)	6.34 (4.44)	5.79 (4.54)	6.57 (4.22)	6.80 (4.48)	.09^a^	.11	.049
CGI-I^b^ score, mean (SD)	4.85 (1.61)	4.68 (1.91)	4.95 (1.36)	5.00 (1.37)	.80^a^	.29	.22
ΔIIEF-5^c^, mean (SD)	3.20 (3.35)	3.37 (3.65)	3.49 (2.96)	2.80 (3.34)	.61^a^	.88	.44
Intention to continue therapy, n/N (%)	192/470 (40.9)	68/180 (37.8)	74/150 (49.3)	50/140 (35.7)	.04^d^	—^e^	—
Actual continuation of therapy, n/N (%)	128/470 (27.2)	47/180 (26.1)	48/150 (32)	33/140 (23.6)	.25^d^	—	—

^a^One-way ANOVA was used for continuous outcomes.

^b^CGI-I: Clinical Global Impression-Improvement.

^c^ΔIIEF-5: change in the 5-item International Index of Erectile Function.

^d^Chi-square tests were used for categorical outcomes.

^e^Not applicable.

### Secondary Outcomes

#### CGI-I

Posttherapy CGI-I data were available for 48.3% (227/470) of participants. Group means were similar (control 4.93, SD 1.60; call 4.95, SD 1.36; SMS 5.08, SD 1.22), corresponding on average to a rating of “minimally improved,” and no between-group differences were detected in unadjusted contrasts (control vs call *P*>.99; control vs SMS *P*>.99; omnibus *F*_2,224_=0.220; *P*=.80). In models adjusted for age, BMI, smoking status, baseline pharmacotherapy, and enrollment-week fixed effects, the group term remained nonsignificant. Further details are available under “Clinical: CGI-I (Adjusted with Covariates)” in Multimedia Appendix 4.

#### ΔIIEF

ΔIIEF data were available for 27% (127/470) of participants, limiting statistical power. The mean change was 3.20 (SD 3.35; range −7 to +13). Group means did not differ (omnibus ANOVA *F*_2,124_=0.503; *P*=.61; Bonferroni-adjusted pairwise comparisons, all *P*≥.99). Consistent with this finding, an ANCOVA of week-12 IIEF-5 adjusted for baseline IIEF-5, age, BMI, smoking status, and baseline pharmacotherapy showed no group effect. Further details are available under “Clinical: IIEF-5 Week 12 (ANCOVA)” in Multimedia Appendix 4.

#### Intention to Continue Therapy (Binary)

Overall, 40.9% (192/470) of participants indicated an intention to continue. By group, 37.8% (68/180) were in the control group, 49.3% (74/150) were in the call group, and 35.7% (50/140) were in the SMS group. An unadjusted omnibus chi-square test showed a group difference in intention to continue (*χ*^2^_2_=6.7; *P*=.04). In the adjusted logistic regression model, the call group vs the control group showed higher odds of intention to continue (OR 1.61, 95% CI 1.03-2.50; *P*=.04), whereas SMS vs control was not significant. Overall model fit was modest (model *χ*^2^_6_=11.7; *P*=.07; low pseudo-*R*^2^), so this result should be interpreted as a small adjusted difference. Exploratory adjusted logistic regression models are reported in the section “Intention to Continue Therapy - Logistic Regression (Adjusted)” in Multimedia Appendix 4.

#### Actual Continuation of Therapy (Conversion)

Overall, 27.2% (128/470) of participants continued therapy. By group, 26.1% (47/180) were in the control group, 32% (48/150) were in the call group, and 23.6% (33/140) were in the SMS group. An unadjusted omnibus chi-square test showed no group difference (*χ*^2^_2_=2.8; *P*=.25).

#### Exploratory Correlations

Exploratory bivariate correlations are reported in the section “Correlations: Active Weeks With Baseline and Clinical Variables” in Multimedia Appendix 4. Adherence correlated negatively with BMI (*r*=−0.123; *P*=.007) and current smoking (*r*=−0.112; *P*=.02). Correlations with age, baseline pharmacotherapy, and baseline IIEF-5 were small, and the correlation with ΔIIEF was not significant (*r*=0.020; *P*=.82).

## Discussion

### Principal Findings

For the primary adherence end point (active weeks, defined as ≥1 completed session per week), unadjusted means favored both support groups (control 5.79; call 6.57; SMS 6.80). The prespecified SMS vs control contrast reached *P*=.049 with a small effect (Cohen *d*=0.22), whereas the call vs control contrast was not significant. In models adjusted for age, BMI, smoking status, and baseline pharmacotherapy, between-group differences were not statistically significant, and the SMS advantage was attenuated. In a calendar-adjusted sensitivity model, the omnibus group term reached significance, yet Bonferroni-adjusted pairwise contrasts vs the control group did not; effect sizes were small. Together with the null findings for the clinical end points, this supports our interpretation that the support strategies primarily aid reactivation after lapses rather than materially increasing the weekly dose. Sensitivity analyses using stricter adherence definitions (≥2 sessions per week or ≥30 minutes of cardiovascular exercise; multidomain weekly goals; thresholds of ≥6, ≥8, and ≥9 active weeks) led to the same conclusion: small or null adjusted differences across groups.

For the mechanistic outcomes, any reactivation after an inactive week occurred more often in the intervention groups than in the control group (omnibus chi-square *P*=.008), indicating that outreach was associated with a higher likelihood of at least 1 return to use after inactivity. Persistence (time to first inactivity) did not differ by group (log-rank *P*=.36).

Clinical secondary outcomes were null overall: adjusted analyses of CGI-I and week-12 IIEF-5 showed no group effects, and the SMS group did not differ from the control group on these end points. Concurrent pharmacotherapy neither explained the engagement signals (active weeks and any reactivation) nor modified the intervention effects; interaction tests by baseline pharmacotherapy status were nonsignificant. Given the self-reported nature of the pharmacotherapy data and the lack of dose and timing information, these analyses are exploratory.

Taken together, these end point results suggest that the mechanics of the support strategy worked as intended at the event level: nudges helped break inactivity spells (ie, reactivation) while producing only small changes in week-to-week adherence totals. In addition, the telephone intervention increased stated intention to continue therapy vs the control group (n/N, 49% vs n/N, 38%; *P*=.04; small effect). Prior evidence indicates that personal contact can strengthen perceived support and reduce uncertainty, which may explain the greater stated willingness to continue [22]. In the real-world pathway studied, continuation typically requires renewed physician involvement after 3 months; thus, stated intention to continue therapy functions as a pragmatic implementation outcome and a proximal indicator of readiness to pursue maintenance treatment. The absence of between-group differences in documented therapy continuation within 3 months likely reflects system-level constraints (eg, access and scheduling) that can decouple motivation from realized continuation [23]. Finally, covariate associations aligned with prior work: higher BMI and current smoking were independently associated with fewer active weeks, underscoring the relevance of lifestyle factors for digital engagement [24].

Overall, the interventions modestly influenced weekly adherence, successfully reactivated some users after inactivity, and, especially for telephone contact, increased motivation to continue therapy [22-27].

### Hypotheses Revisited

We prespecified that inactivity-triggered supports would increase weekly engagement (H1), that telephone support might increase stated intention to continue therapy (H2), and that clinical outcomes could differ between groups (H3, exploratory). The results supported H1 in a mechanism-aligned way: the support groups, particularly the SMS group, showed higher odds of any reactivation after ≥7 days of inactivity, with small gains in active weeks; however, within-week dose did not materially increase. H2 was supported: the telephone group reported a higher intention to continue therapy at week 12. H3 was not supported: IIEF-5 and CGI-I did not differ between groups. Because IIEF-5 was collected only at the end of the intervention among users who remained engaged, we consider the clinical outcomes exploratory and include a nonresponder analysis (baseline responder vs nonresponder contrasts and a logistic regression model of follow-up completion with covariates and study group; the group term was not associated with completion). Given the short 12-week follow-up and the quasi-experimental design, we interpret the observed effects as engagement-focused (preventing dropout through weekly reactivation) rather than evidence of therapeutic efficacy and therefore avoid causal claims.

### Comparison With Prior Work

Prior research has shown that digital reminders, especially SMS messages, can support adherence across chronic conditions, including type 2 diabetes and cardiovascular disease [6-8]. Meta-analytic and trial evidence similarly point to mobile prompts as practical “nudges” for health behavior change [24,26-29]. This study extends these principles to a sexual medicine setting, where adherence involves completing exercise-based therapeutic sessions rather than taking medication. In our data, the SMS group showed a nominal improvement in active weeks in the unadjusted analysis, but adjusted analyses indicated small, nonsignificant differences vs the control group. This pattern is consistent with the broader literature, in which effect sizes for digital nudges are often modest and context-dependent [6-8,28,29].

At the same time, not all large, pragmatic evaluations have found benefits. A recent randomized trial by Ho et al [30] testing reminder and nudge strategies and personalized data feedback for chronic cardiovascular medications did not improve adherence (proportion of days covered) relative to usual care, underscoring how clinical context, target behavior, and implementation details shape effectiveness.

Finally, our observation that telephone support increased stated intention to continue therapy complements prior work on the motivational value of personal contact, while the absence of a corresponding increase in documented therapy continuation highlights a well-described intention-behavior gap in health care [31]. Together, these findings suggest that scalable digital nudges may help restart engagement after lapses, whereas relational outreach can shift motivation. Both are relevant but may be insufficient on their own without system-level supports that translate motivation into sustained behavior.

### Strengths and Limitations

This evaluation was conducted within routine operations, using a rotating calendar-week schedule to deliver SMS and telephone outreach in the same workflow patients encounter. That pragmatic embedding enhances external validity and supports scalability. We also aligned the outcomes with the intervention’s mechanism of action, prioritizing the proximal ability of outreach to restart use after a ≥7-day lapse and summarizing week-to-week participation as “active weeks” (≥1 completed session). Because adherence was derived from app telemetry with enforced completion rules, recorded sessions reflect purposeful therapeutic activity rather than passive app use. Finally, the cohort of 470 men with physician-diagnosed ED was clinically relevant and well characterized; baseline profiles were broadly comparable across groups, and prespecified covariates (age, BMI, smoking status, and baseline pharmacotherapy) were accounted for in the adjusted analyses.

This study used a quasi-experimental allocation by calendar week rather than individual randomization. Although this approach facilitated implementation in a real-world setting and minimized contamination between participants, it introduces potential biases, including unmeasured confounding and time-related effects. Although we adjusted for covariates and examined calendar-time sensitivity, residual and time-varying confounding cannot be excluded, and the design was unblinded. Industry collaboration was limited to the design of reminders and, despite safeguards (independent statistical cross-checks, governance procedures, conflict-of-interest disclosures, and planned code and data sharing), had no influence on data analysis, interpretation, or reporting. Clinical end points were limited by voluntary follow-up in routine care (IIEF-5 and CGI-I), which reduced statistical power and raised the possibility of missing-not-at-random mechanisms; nonresponder analyses did not indicate between-group differences in follow-up, but the clinical findings are interpreted as exploratory. Log-based adherence measures, while objective, do not capture training quality or within-week dose beyond completion thresholds, and passive engagement is not observed; active weeks therefore summarize participation but may underestimate intensity. The observation window was 12 weeks, so durability is unknown. Notably, the higher stated intention to continue therapy after telephone outreach did not translate into higher documented therapy continuation within 3 months, plausibly reflecting system-level constraints (eg, access, scheduling, and represcription). The single-item intention measure is susceptible to response and social desirability bias and should be viewed as an implementation indicator rather than a clinical outcome. Baseline pharmacotherapy was self-reported without dose or timing verification; interaction tests did not suggest effect modification by medication status, but these analyses remain exploratory. Finally, generalizability may be limited because the findings arise from a single DiGA within Germany’s reimbursement and 12-week prescription context and may not extend to other health care systems, payment models, or digital therapeutics with different content or support workflows; replication with longer follow-up is warranted.

### Implications for Clinical Practice and Future Research

Taken together, our findings suggest that inactivity-triggered outreach can reliably restart engagement after lapses, whereas the sustained week-to-week dose increased only modestly and did not remain significant after covariate adjustment. The interventions were associated with a higher probability of reactivation following an inactive week, and telephone outreach was additionally associated with a greater stated intention to continue therapy at 12 weeks; by contrast, adjusted differences in total active weeks were small, and clinical end points (IIEF-5 and CGI-I) did not differ between groups. In practice, automated SMS reminders are best used as a scalable first-line nudge to break ≥7-day inactivity spells, aligning support with the mechanism most clearly affected (ie, reactivation). Telephone outreach should be reserved for escalation, targeting patients who remain inactive after SMS or who are at higher risk of disengagement, where it may enhance motivation to continue therapy. A pragmatic approach is to combine and sequence support strategies (SMS followed by telephone outreach if the patient remains inactive), integrate them with appointment scheduling or represcription workflows, and continuously monitor barriers to therapy continuation (eg, access and logistics) that can decouple motivation from realized continuation. Expectations for clinical impact should be calibrated accordingly: within the 12-week observation period, we observed no between-group differences in clinical outcomes; therefore, these support strategies should be positioned primarily as engagement tools. Any downstream clinical benefits will likely require longer follow-up and/or stronger changes in dose intensity. In short, support strategies modestly influenced weekly adherence totals but were most effective at reactivating use after lapses, with telephone contact additionally increasing motivation; programs should therefore emphasize preventing dropout at the event level and apply stepped, risk-targeted escalation while avoiding overinterpretation of short-term adherence changes as clinical efficacy.

### Conclusions

In this pragmatic, calendar-time–allocated evaluation of a digital ED therapy, inactivity-triggered support showed mechanism-aligned effects on engagement. Across groups, outreach was associated with a higher probability of any reactivation after an inactive week (ie, breaking inactivity spells). In addition, the telephone intervention yielded a higher stated intention to continue therapy at 12 weeks vs the control group, a pragmatic implementation outcome in settings where continuation typically requires renewed prescription. By contrast, differences in weekly adherence totals (active weeks) were small and not statistically significant after covariate adjustment, and clinical end points (IIEF-5 and CGI-I) did not differ between groups over 12 weeks.

Taken together, these findings support a practical division of labor:

- Automated SMS reminders as a scalable first-line nudge to prevent dropout by reactivating users after lapses; and
- Telephone outreach as a more resource-intensive escalation that may shift motivation (higher intention to continue).

These are engagement tools, not stand-alone efficacy treatments. Any downstream clinical benefit will likely depend on sustained exposure and system factors (eg, access to follow-up care) that translate motivation into realized continuation. The promise of simple, low-cost digital communication in routine care remains noteworthy [32], but effect sizes were modest, and causal inference is limited by the quasi-experimental design.

Future work should test stepped, adaptive support (eg, SMS reminders followed by telephone outreach for persistent inactivity), optimize timing and targeting, address conversion bottlenecks (scheduling and represcription), and evaluate longer-term clinical outcomes and cost-effectiveness. Replication in other health systems and with alternative digital therapeutics will clarify generalizability and help define where, for whom, and at what intensity digital nudges deliver the most value.

## Appendix

Multimedia Appendix 1 Ethics approval document.

Multimedia Appendix 2 Contraindications and precautions: with ICD-10 (International Classification of Diseases, Tenth Revision) codes.

Multimedia Appendix 3 Week-by-week overview of module content in the app.

Multimedia Appendix 4 SPSS Syntax for Analysis.

## Abbreviations

ANCOVA: analysis of covariance
CGI-I: Clinical Global Impression-Improvement
DiGA: Digitale Gesundheitsanwendung
DVG: Digitale-Versorgung-Gesetz
ED: erectile dysfunction
GDPR: General Data Protection Regulation
GLM: general linear model
ICD-10: International Statistical Classification of Diseases, Tenth Revision
IIEF-5: 5-item International Index of Erectile Function
MUSE: medicated urethral system for erection
OR: odds ratio
PDE-5: phosphodiesterase type 5
PFMT: pelvic floor muscle training
SKAT: Schwellkörper-Autoinjektionstherapie
WHO: World Health Organization

## Data Availability

The participant-level dataset analyzed during the current study is owned by Kranus Health GmbH and is available from Zenodo under restricted access [33]. Access requires a data-use agreement with the rights holder and is limited to research and teaching; no reidentification or redistribution is permitted. The full analysis code (SPSS version 30) and variable codebook are openly available at Zenodo [34].
