# Supplementary material for: Support Strategies to Enhance Adherence to a Prescription Digital Therapeutic for Erectile Dysfunction: Retrospective Quasi-Experimental Cohort Study
Source: JMIR Mhealth Uhealth. 2026 Jul 14;14:e76724. doi: 10.2196/76724 (PMC13416304; doi:10.2196/76724)
Supplement: Multimedia Appendix 1 [file mhealth_v14i1e76724_app1.pdf]

Study Title:
The Impact of Technology-Based Interventions on Adherence in Digital Therapy for Erectile Dysfunction (Acronym: INTERADIG)

Ethics Committee Approval
Institutional Ethics Committee of Charité – Universitätsmedizin Berlin
Application Number: EA4/104/24
Approval Date: December 16, 2024

Document Summary:
This appendix includes the original ethics committee correspondence confirming the approval of the study protocol submitted on December 10, 2024. The ethics application (version dated December 10, 2024) was reviewed and accepted. The committee confirmed that all conditions outlined in the initial review have been fulfilled.

Language Note:
The attached correspondence is provided in the original German language.
