# Supplementary material for: Support Strategies to Enhance Adherence to a Prescription Digital Therapeutic for Erectile Dysfunction: Retrospective Quasi-Experimental Cohort Study
Source: JMIR Mhealth Uhealth. 2026 Jul 14;14:e76724. doi: 10.2196/76724 (PMC13416304; doi:10.2196/76724)
Supplement: Multimedia Appendix 2 [file mhealth_v14i1e76724_app2.pdf]

Multimedia Appendix 11 - Contraindications and Precautions (with ICD‑10 Codes)

This appendix lists inclusion criteria and clinical exclusions for participation in the digital therapeutic program. Contraindications were derived from the provider's instructions for use (IFU) and standard cardiovascular safety guidance for exercise‑based rehabilitation. ICD‑10 codes are provided to facilitate screening/documentation; exact subcodes may vary by national adaptation (ICD‑10‑GM vs ICD‑10‑CM).

Inclusion criteria

Criterion	Notes / Codes
Diagnosed erectile dysfunction	ICD‑10: N52.*
Age ≥ 18 years	Adults only
IIEF‑5 score ≤ 21	Validated ED severity threshold

Exclusion criteria

Grouped into absolute and relative contraindications. Relative items require physician judgment on risk–benefit.

A. Absolute contraindications

Condition	ICD‑10 example	Clarification
Unstable angina pectoris	I20.0	Exclude until clinically stabilized.
Recent myocardial infarction (29 days to <4 months post‑event)	I25.2 (old MI) with event date noted; prior acute MI I21.* / I22.*	Acute MI is coded I21.* for ≤28 days. Beyond 28 days, code sequelae as I25.2 and document recency; require cardiology clearance.

Absolute contraindications without ICD‑10 codes: none.

B. Relative contraindications (with ICD-10 examples) - prescriber to assess

Condition	Threshold / definition	ICD-10 example(s)	Notes
Hypertension exceeding 170/100 mmHg	Resting SBP/DBP persistently >170/100	I10	Defer until better controlled; consider I11.* if cardiac involvement.
Left ventricular dysfunction / heart failure (NYHA III–IV)	NYHA III–IV or decompensated HF	I50.*	Record NYHA class; optimize therapy before enrollment.
Cardiomyopathies	Dilated, hypertrophic, restrictive	I42.*	Assess stability; consider cardiology input.
Stable or moderate angina pectoris	Symptoms with exertion despite therapy	I20.* (excl. I20.0)	Upgrade to absolute if unstable/accelerating.
Valvular heart disease (moderate–severe)	Hemodynamically significant stenosis/regurgitation	I34–I39	Individualize by valve/severity; cardiology input.
High-risk cardiac arrhythmias	Sustained VT; poorly controlled AF; syncope	I47.*; I48.*; I49.*	Exclude until rhythm/rate control achieved.
Diabetes mellitus with poor control/complications	e.g., very high HbA1c; autonomic neuropathy	E10.* / E11.*	Assess CV risk before exercise escalation.
Cerebrovascular disease (recent)	Stroke/TIA within 6 weeks	I63.*; I61.*; G45.*	Temporary exclusion; clearance after 6 weeks.
Recent myocardial infarction (very recent)	<6 weeks since event	I21.* / I22.*	Temporary exclusion; follow local rehab policy.

C. Relative contraindications without specific ICD-10 codes - prescriber to assess

Clinical factor	Clarification / examples
>3 cardiovascular risk factors	Examples: hypertension >150/90, diabetes, dyslipidemia, smoking/tobacco use, physical inactivity, obesity (code individually as applicable: I10, E10/E11, E78.*, F17.2, Z72.3, E66.*).
Inability to physically participate	Mobility limitations or acute conditions that make participation unsafe; document rationale.

Notes:

1) Time windows (e.g., 29 days–<4 months post-MI; 6-week blackout after MI or stroke) reflect program safety policy; prescribers may individualize per current status and guidelines.

2) ICD-10 subcategories differ by national variant (ICD-10-GM vs ICD-10-CM). Use local coding manuals; where subcodes are uncertain, three-character categories (e.g., I50.*) with clinical qualifiers (e.g., NYHA class) are acceptable.

3) This appendix supports safety screening. Clinical judgment prevails.
