# Supplementary material for: Support Strategies to Enhance Adherence to a Prescription Digital Therapeutic for Erectile Dysfunction: Retrospective Quasi-Experimental Cohort Study
Source: JMIR Mhealth Uhealth. 2026 Jul 14;14:e76724. doi: 10.2196/76724 (PMC13416304; doi:10.2196/76724)
Supplement: Multimedia Appendix 3 [file mhealth_v14i1e76724_app3.pdf]

Multimedia Appendix 7 - Week-by-Week Module Overview

This appendix summarizes the 12-week program structure used in the digital therapeutic. It outlines typical weekly components, nominal durations, and example tasks. Durations are indicative and may be individualized based on the initial health questionnaire and clinical context. Cardiovascular activities are patient-selected (e.g., brisk walking, jogging, cycling). Pelvic-floor training (PFMT) combines short daily contraction sets with a weekly physiotherapy task. Mindfulness/relaxation content is provided as guided audio, and sex-therapy content (e.g., sensate-focus–based exercises) begins in Week 4. Educational content consists of brief daily articles.

Nominal total program dose (12 weeks):

• Cardiovascular endurance: 18 hours total (approx. 90 minutes/week, typically two 45-minute sessions).

• Pelvic-floor strengthening (PFMT + physiotherapy): 3.5 hours total.

• Mental/relaxation and sex-therapy content: 3.5 hours total (approx. 10 minutes/week; sex-therapy content from Week 4 onward).

• Educational content: 1.5 hours total (short daily readings).

Week	Cardiovascular endurance (target & examples)	Pelvic–floor training (PFMT) & physiotherapy	Mental / Sex-therapy content	Educational topic (example)	Expected weekly time (approx.)
Week 1	Moderate aerobic training ≥2×45 min/week (choose: brisk walking, jogging, cycling). Use interval or steady-state plans adapted to baseline fitness.	Daily PFMT micro-sessions (3–10 min; contraction sets with cues) + 1 weekly physiotherapy task (5–10 min; pelvic-floor/hip/trunk mobility & control).	1 guided relaxation/mindfulness audio (10 min): e.g., breathing, body-scan, tension-release.	Principles and causes of erectile dysfunction	Approx. 2.3-2.7 h (90 min cardio + 35-45 min PFMT/physio + 10 min mental + brief readings)

Week 2	Moderate aerobic training ≥2×45 min/week (choose: brisk walking, jogging, cycling). Use interval or steady-state plans adapted to baseline fitness.	Daily PFMT micro-sessions (3-10 min; contraction sets with cues) + 1 weekly physiotherapy task (5-10 min; pelvic-floor/hip/trunk mobility & control).	1 guided relaxation/mindfulness audio (10 min): e.g., breathing, body-scan, tension-release.	What is the pelvic floor?	Approx. 2.3-2.7 h (90 min cardio + 35-45 min PFMT/physio + 10 min mental + brief readings)
Week 3	Moderate aerobic training ≥2×45 min/week (choose: brisk walking, jogging, cycling). Use interval or steady-state plans adapted to baseline fitness.	Daily PFMT micro-sessions (3-10 min; contraction sets with cues) + 1 weekly physiotherapy task (5-10 min; pelvic-floor/hip/trunk mobility & control).	1 guided relaxation/mindfulness audio (10 min): e.g., breathing, body-scan, tension-release.	Diet and erectile dysfunction	Approx. 2.3-2.7 h (90 min cardio + 35-45 min PFMT/physio + 10 min mental + brief readings)
Week 4	Moderate aerobic training ≥2×45 min/week (choose: brisk walking, jogging, cycling). Use interval or steady-state plans adapted to baseline fitness.	Daily PFMT micro-sessions (3-10 min; contraction sets with cues) + 1 weekly physiotherapy task (5-10 min; pelvic-floor/hip/trunk mobility & control).	1 guided relaxation (10 min) + 1 sex-therapy audio (10 min): e.g., sensate-focus, self-acceptance, body exploration.	Mind and erectile dysfunction	Approx. 2.7-3.0 h (90 min cardio + 35-45 min PFMT/physio + 20 min mental/sex-therapy + brief readings)

Week 5	Moderate aerobic training ≥2×45 min/week (choose: brisk walking, jogging, cycling). Use interval or steady-state plans adapted to baseline fitness.	Daily PFMT micro-sessions (3-10 min; contraction sets with cues) + 1 weekly physiotherapy task (5-10 min; pelvic-floor/hip/trunk mobility & control).	1 guided relaxation (10 min) + 1 sex-therapy audio (10 min): e.g., sensate-focus, self-acceptance, body exploration.	Habits and motivation	Approx. 2.7-3.0 h (90 min cardio + 35-45 min PFMT/physio + 20 min mental/sex-therapy + brief readings)
Week 6	Moderate aerobic training ≥2×45 min/week (choose: brisk walking, jogging, cycling). Use interval or steady-state plans adapted to baseline fitness.	Daily PFMT micro-sessions (3-10 min; contraction sets with cues) + 1 weekly physiotherapy task (5-10 min; pelvic-floor/hip/trunk mobility & control).	1 guided relaxation (10 min) + 1 sex-therapy audio (10 min): e.g., sensate-focus, self-acceptance, body exploration.	Treatment options for erectile dysfunction	Approx. 2.7-3.0 h (90 min cardio + 35–45 min PFMT/physio + 20 min mental/sex-therapy + brief readings)
Week 7	Moderate aerobic training ≥2×45 min/week (choose: brisk walking, jogging, cycling). Use interval or steady-state plans adapted to baseline fitness.	Daily PFMT micro-sessions (3-10 min; contraction sets with cues) + 1 weekly physiotherapy task (5-10 min; pelvic-floor/hip/trunk mobility & control).	1 guided relaxation (10 min) + 1 sex-therapy audio (10 min): e.g., sensate-focus, self-acceptance, body exploration.	Erectile dysfunction and other diseases	Approx. 2.7-3.0 h (90 min cardio + 35-45 min PFMT/physio + 20 min mental/sex-therapy + brief readings)

Week 8	Moderate aerobic training ≥2×45 min/week (choose: brisk walking, jogging, cycling). Use interval or steady-state plans adapted to baseline fitness.	Daily PFMT micro-sessions (3-10 min; contraction sets with cues) + 1 weekly physiotherapy task (5-10 min; pelvic-floor/hip/trunk mobility & control).	1 guided relaxation (10 min) + 1 sex-therapy audio (10 min): e.g., sensate-focus, self-acceptance, body exploration.	Additional benefits of physical activity	Approx. 2.7-3.0 h (90 min cardio + 35-45 min PFMT/physio + 20 min mental/sex-therapy + brief readings)
Week 9	Moderate aerobic training ≥2×45 min/week (choose: brisk walking, jogging, cycling). Use interval or steady-state plans adapted to baseline fitness.	Daily PFMT micro-sessions (3-10 min; contraction sets with cues) + 1 weekly physiotherapy task (5-10 min; pelvic-floor/hip/trunk mobility & control).	1 guided relaxation (10 min) + 1 sex-therapy audio (10 min): e.g., sensate-focus, self-acceptance, body exploration.	Benefits of pelvic-floor rehabilitation	Approx. 2.7-3.0 h (90 min cardio + 35-45 min PFMT/physio + 20 min mental/sex-therapy + brief readings)
Week 10	Moderate aerobic training ≥2×45 min/week (choose: brisk walking, jogging, cycling). Use interval or steady-state plans adapted to baseline fitness.	Daily PFMT micro-sessions (3-10 min; contraction sets with cues) + 1 weekly physiotherapy task (5-10 min; pelvic-floor/hip/trunk mobility & control).	1 guided relaxation (10 min) + 1 sex-therapy audio (10 min): e.g., sensate-focus, self-acceptance, body exploration.	Couple relationship and communication	Approx. 2.7-3.0 h (90 min cardio + 35-45 min PFMT/physio + 20 min mental/sex-therapy + brief readings)

Week 11	Moderate aerobic training ≥2×45 min/week (choose: brisk walking, jogging, cycling). Use interval or steady-state plans adapted to baseline fitness.	Daily PFMT micro-sessions (3-10 min; contraction sets with cues) + 1 weekly physiotherapy task (5-10 min; pelvic-floor/hip/trunk mobility & control).	1 guided relaxation (10 min) + 1 sex-therapy audio (10 min): e.g., sensate-focus, self-acceptance, body exploration.	Facts and figures on men's health	Approx. 2.7-3.0 h (90 min cardio + 35-45 min PFMT/physio + 20 min mental/sex-therapy + brief readings)
Week 12	Moderate aerobic training ≥2×45 min/week (choose: brisk walking, jogging, cycling). Use interval or steady-state plans adapted to baseline fitness.	Daily PFMT micro-sessions (3-10 min; contraction sets with cues) + 1 weekly physiotherapy task (5-10 min; pelvic-floor/hip/trunk mobility & control).	1 guided relaxation (10 min) + 1 sex-therapy audio (10 min): e.g., sensate-focus, self-acceptance, body exploration.	What does prevention mean?	Approx. 2.7-3.0 h (90 min cardio + 35-45 min PFMT/physio + 20 min mental/sex-therapy + brief readings)

Notes:

1) Durations are nominal targets; actual plans are individualized based on the initial health questionnaire and clinician guidance.

2) PFMT sessions use app-guided timers and completion rules; physiotherapy tasks emphasize pelvic-floor control and adjacent core/hip mobility.

3) Sex-therapy content is introduced from Week 4 (e.g., sensate-focus exercises) alongside ongoing mindfulness/relaxation.

4) Educational articles are brief; topics rotate daily to reinforce behavior change and understanding of ED and related risk factors.
