# Supplementary material for: Support Strategies to Enhance Adherence to a Prescription Digital Therapeutic for Erectile Dysfunction: Retrospective Quasi-Experimental Cohort Study
Source: JMIR Mhealth Uhealth. 2026 Jul 14;14:e76724. doi: 10.2196/76724 (PMC13416304; doi:10.2196/76724)
Supplement: Multimedia Appendix 4 [file mhealth_v14i1e76724_app4.pdf]

```
* ============================================================================.
* INTERADIG SYNTAX.
* Date: 2025-11-10.
* ============================================================================.

SET PRINTBACK=OFF MPRINT=ON OLANG=ENGLISH.
SET DECIMAL=DOT.

* ============================================================================.
* SECTION 0: VERIFY GROUP VARIABLE.
* ============================================================================.

TITLE 'Data Setup: Group Variable'.

* Setup: ensure no splits/filters and readable labels for gruppe.
SPLIT FILE OFF.
FILTER OFF.
EXECUTE.

* Apply variable label and value labels to gruppe.
VARIABLE LABELS gruppe "Group".
VALUE LABELS gruppe
   1 'Control'
   2 'Call'
   3 'SMS'.
VARIABLE LEVEL gruppe (NOMINAL).
FORMATS age bmi iief_week_1 iief_week_12 (F8.2).
FORMATS Active_weeks (F8.2).
VARIABLE LEVEL Active_weeks (SCALE).
EXECUTE.

* Verify all three groups present.
FREQUENCIES VARIABLES=gruppe.

* Remove any filters.
FILTER OFF.
SPLIT FILE OFF.
USE ALL.
EXECUTE.

* ============================================================================.
* SECTION 1: BASELINE PHARMACOTHERAPY.
* ============================================================================.
```

```
TITLE 'Baseline: Pharmacotherapy'.

COMPUTE any_pharma = 0.
IF (takes_pde5 = 1 OR takes_skat_or_muse = 1) any_pharma = 1.
VARIABLE LABELS any_pharma "Baseline: Any ED pharmacotherapy".
VALUE LABELS any_pharma 0 "No" 1 "Yes".
EXECUTE.

* ===============================================================.
* SECTION 2: BASELINE CHARACTERISTICS.
* ===============================================================.

TITLE 'Baseline Characteristics: Continuous Variables (N=470)'.

MEANS TABLES=age bmi iief_week_1 BY gruppe
  /CELLS MEAN STDDEV COUNT.

* Replace ONEWAY with GLM for age.
TITLE 'Baseline: Age by Group (GLM with Bonferroni)'.
GLM age BY gruppe
  /METHOD=SSTYPE(3)
  /EMMEANS=TABLES(gruppe) COMPARE ADJ(BONFERRONI)
  /PRINT=DESCRIPTIVE ETASQ HOMOGENEITY
  /CRITERIA=ALPHA(.05)
  /DESIGN=gruppe.

* Replace ONEWAY with GLM for BMI.
TITLE 'Baseline: BMI by Group (GLM with Bonferroni)'.
GLM bmi BY gruppe
  /METHOD=SSTYPE(3)
  /EMMEANS=TABLES(gruppe) COMPARE ADJ(BONFERRONI)
  /PRINT=DESCRIPTIVE ETASQ HOMOGENEITY
  /CRITERIA=ALPHA(.05)
  /DESIGN=gruppe.

* Replace ONEWAY with GLM for baseline IIEF.
TITLE 'Baseline: IIEF Week 1 by Group (GLM with Bonferroni)'.
GLM iief_week_1 BY gruppe
  /METHOD=SSTYPE(3)
  /EMMEANS=TABLES(gruppe) COMPARE ADJ(BONFERRONI)
  /PRINT=DESCRIPTIVE ETASQ HOMOGENEITY
  /CRITERIA=ALPHA(.05)
  /DESIGN=gruppe.
```

```
TITLE 'Baseline Characteristics: Categorical Variables'.

CROSSTABS /TABLES=gruppe BY is_smoker
   /STATISTICS=CHISQ
   /CELLS=COUNT ROW COLUMN.

CROSSTABS /TABLES=gruppe BY any_pharma
   /STATISTICS=CHISQ
   /CELLS=COUNT ROW COLUMN.

* ==============================================================.
* SECTION 3: ADHERENCE METRICS.
* ==============================================================.

TITLE 'Adherence Metrics'.

COMPUTE total_trainings = SUM(trainings_week_1 TO trainings_week_12).
VARIABLE LABELS total_trainings "Total training sessions".
EXECUTE.

DO REPEAT w = trainings_week_1 TO trainings_week_6
        / wnz = train_non0_1 TO train_non0_6.
  COMPUTE wnz = w.
  IF (w = 0) wnz = $SYSMIS.
END REPEAT.
DO REPEAT w = trainings_week_7 TO trainings_week_12
        / wnz = train_non0_7 TO train_non0_12.
  COMPUTE wnz = w.
  IF (w = 0) wnz = $SYSMIS.
END REPEAT.
COMPUTE median_trainings_active_week = MEDIAN(train_non0_1 TO train_non0_12).
VARIABLE LABELS median_trainings_active_week "Median trainings per active week".
FORMAT median_trainings_active_week (F8.2).
EXECUTE.

COMPUTE Active_weeks = (trainings_week_1 > 0) + (trainings_week_2 > 0) +
(trainings_week_3 > 0)
                    + (trainings_week_4 > 0) + (trainings_week_5 > 0) + (trainings_week_6 > 0)
                    + (trainings_week_7 > 0) + (trainings_week_8 > 0) + (trainings_week_9 > 0)
                    + (trainings_week_10 > 0) + (trainings_week_11 > 0) + (trainings_week_12 > 0).
VARIABLE LABELS Active_weeks "Active weeks (≥1 session per week, 0-12)".
EXECUTE.

COMPUTE engagement_ratio = Active_weeks / 12.
```

```
VARIABLE LABELS engagement_ratio "Engagement ratio".
FORMAT engagement_ratio (F4.2).
EXECUTE.

* ===============================================================.
* SECTION 4: PRIMARY OUTCOME - ACTIVE WEEKS (UNADJUSTED).
* ===============================================================.

TITLE 'PRIMARY OUTCOME: Active Weeks (Unadjusted - GLM with Bonferroni)'.

MEANS TABLES=Active_weeks BY gruppe
  /CELLS MEAN STDDEV COUNT.

* Replace ONEWAY with GLM.
GLM Active_weeks BY gruppe
  /METHOD=SSTYPE(3)
  /EMMEANS=TABLES(gruppe) COMPARE ADJ(BONFERRONI)
  /PRINT=DESCRIPTIVE ETASQ HOMOGENEITY
  /CRITERIA=ALPHA(.05)
  /DESIGN=gruppe.

* ===============================================================.
* SECTION 4B: UNADJUSTED PLANNED CONTRASTS FOR ACTIVE WEEKS.
* ===============================================================.

TITLE 'PRIMARY OUTCOME: Active Weeks - Unadjusted Pairwise Comparisons'.

* Unadjusted pairwise t-tests for Active_weeks (two-sample, 2-sided).
* Includes gruppe means, SDs, 95% CI, Levene's test for equality of variances.

* 1) Control vs Call.
T-TEST
  GROUPS = gruppe(1 2)
  /VARIABLES = Active_weeks
  /MISSING = ANALYSIS
  /CRITERIA = CI(.95).

* 2) Control vs SMS.
T-TEST
  GROUPS = gruppe(1 3)
  /VARIABLES = Active_weeks
  /MISSING = ANALYSIS
  /CRITERIA = CI(.95).
```

```
* 3) Call vs SMS.
T-TEST
  GROUPS = gruppe(2 3)
  /VARIABLES = Active_weeks
  /MISSING = ANALYSIS
  /CRITERIA = CI(.95).

* ===================================================================.
* SECTION 4C: FIGURE 1 - BOXPLOT OF ACTIVE WEEKS BY GROUP.
* ===================================================================.

TITLE 'FIGURE 1: Distribution of Active Training Weeks by Group'.

EXAMINE VARIABLES=Active_weeks BY gruppe
  /PLOT=BOXPLOT
  /STATISTICS=NONE
  /NOTOTAL
  /MISSING=LISTWISE.

* ===================================================================.

* ===================================================================.
* SECTION 5: PRIMARY OUTCOME - ACTIVE WEEKS (ADJUSTED).
* ===================================================================.

TITLE 'PRIMARY OUTCOME: Active Weeks (Standard Covariate Adjustment)'.

* Standard adjustment WITHOUT enrollment time.
GLM Active_weeks BY gruppe
  WITH age bmi is_smoker any_pharma
  /METHOD=SSTYPE(3)
  /EMMEANS=TABLES(gruppe) WITH(age=MEAN bmi=MEAN is_smoker=MEAN
any_pharma=MEAN)
          COMPARE ADJ(BONFERRONI)
  /PRINT=ETASQ PARAMETER DESCRIPTIVE
  /CRITERIA=ALPHA(.05)
  /DESIGN=age bmi is_smoker any_pharma gruppe.

* ===================================================================.
* SECTION 5B: PRIMARY OUTCOME - CALENDAR-TIME ADJUSTED.
* ===================================================================.

TITLE 'PRIMARY OUTCOME: Active Weeks (Calendar-Time Sensitivity Analysis)'.
```

```
-------------------------.
* STEP 1: CREATE ENROLLMENT TIME VARIABLE.
* --------------------------------------------------------------.

* If start_date is already an SPSS date number, simply copy it:
COMPUTE enrollment_date = start_date.
VARIABLE LABELS enrollment_date "Enrollment date (SPSS date)".
FORMATS enrollment_date (SDATE10).
EXECUTE.

* Verify the dates look correct (should show actual dates like 2022-2024).
FREQUENCIES VARIABLES=enrollment_date
  /FORMAT=NOTABLE
  /STATISTICS=MEAN STDDEV MIN MAX.

* Alternative: If the above STILL shows huge numbers, then start_date is in seconds
* and we need to keep it as-is without any formatting:
* COMPUTE enrollment_date = start_date.
* EXECUTE.

* Create days since first enrollment.
AGGREGATE OUTFILE=* MODE=ADDVARIABLES OVERWRITE=YES
  /BREAK=
  /min_date=MIN(enrollment_date).

COMPUTE days_since_start = (enrollment_date - min_date) / 86400.
VARIABLE LABELS days_since_start "Days since first enrollment".
EXECUTE.

* Verify days_since_start is reasonable (should be between 0 and ~1000 for most studies).
DESCRIPTIVES VARIABLES=days_since_start
  /STATISTICS=MEAN STDDEV MIN MAX.

* If max value is > 10000, there's likely a date conversion problem.
* In that case, you may need to check the format of start_date in your data.

* Standardize for regression.
DESCRIPTIVES VARIABLES=days_since_start
  /SAVE.

COMPUTE enrollment_time_z = Zdays_since_start.
VARIABLE LABELS enrollment_time_z "Enrollment time (standardized)".
EXECUTE.
```

```
* Clean up temporary z-score variable.
DELETE VARIABLES Zdays_since_start.
EXECUTE.

* ---------------------------------------------------------------.
* STEP 2: CHECK ENROLLMENT TIMING BY GROUP.
* ---------------------------------------------------------------.

TITLE 'Calendar-Time: Enrollment Timing by Group'.

MEANS TABLES=days_since_start BY gruppe
  /CELLS MEAN STDDEV MIN MAX COUNT.

GLM days_since_start BY gruppe
  /METHOD=SSTYPE(3)
  /EMMEANS=TABLES(gruppe) COMPARE ADJ(BONFERRONI)
  /PRINT=DESCRIPTIVE ETASQ
  /CRITERIA=ALPHA(.05)
  /DESIGN=gruppe.

* ---------------------------------------------------------------.
* STEP 3: BASELINE CHARACTERISTICS VS ENROLLMENT TIME.
* ---------------------------------------------------------------.

TITLE 'Calendar-Time: Baseline Characteristics vs Enrollment Time'.

CORRELATIONS
  /VARIABLES=enrollment_time_z age bmi iief_week_1
  /PRINT=TWOTAIL SIG
  /STATISTICS=DESCRIPTIVES.

* ---------------------------------------------------------------.
* STEP 4: OUTCOMES VS ENROLLMENT TIME.
* ---------------------------------------------------------------.

TITLE 'Calendar-Time: Outcomes vs Enrollment Time'.

CORRELATIONS
  /VARIABLES=enrollment_time_z Active_weeks iief_week_12 cgi_score
  /PRINT=TWOTAIL SIG
  /STATISTICS=DESCRIPTIVES.

* Regression: Active weeks (with enrollment time).
TITLE 'Calendar-Time: Active Weeks Regression'.
```

```
REGRESSION
  /CRITERIA=PIN(.05) POUT(.10)
  /STATISTICS COEFF CI(95) R ANOVA
  /DEPENDENT Active_weeks
  /METHOD=ENTER age bmi is_smoker any_pharma enrollment_time_z.

* ---------------------------------------------------------------.
* STEP 5: PRIMARY ANALYSIS ADJUSTED FOR ENROLLMENT TIME.
* ---------------------------------------------------------------.

TITLE 'PRIMARY OUTCOME: Active Weeks (Adjusted for Covariates + Calendar-Time)'.

GLM Active_weeks BY gruppe
  WITH age bmi is_smoker any_pharma enrollment_time_z
  /METHOD=SSTYPE(3)
  /EMMEANS=TABLES(gruppe) WITH(age=MEAN bmi=MEAN is_smoker=MEAN
                          any_pharma=MEAN enrollment_time_z=MEAN)
         COMPARE ADJ(BONFERRONI)
  /PRINT=ETASQ PARAMETER DESCRIPTIVE
  /CRITERIA=ALPHA(.05)
  /DESIGN=age bmi is_smoker any_pharma enrollment_time_z gruppe.

* ---------------------------------------------------------------.
* STEP 6: TEST GROUP × TIME INTERACTION.
* ---------------------------------------------------------------.

TITLE 'Calendar-Time: Group × Time Interaction Test'.

* Note: Testing if intervention effects vary by enrollment timing.
* Comparing model with vs without group*time interaction.

* Model WITH interaction (separate slopes for each group).
GLM Active_weeks BY gruppe
  WITH age bmi is_smoker any_pharma enrollment_time_z
  /METHOD=SSTYPE(3)
  /PRINT=PARAMETER ETASQ
  /LMATRIX gruppe 1 0 0; gruppe 0 1 0; gruppe 0 0 1
  /DESIGN=age bmi is_smoker any_pharma gruppe enrollment_time_z
        gruppe BY enrollment_time_z.

* =================================================================.
* SECTION 6: GOAL ATTAINMENT.
* =================================================================.
```

```
TITLE 'Goal Attainment by Domain'.

DO REPEAT pfw = pelvic_floor_target_progress_week_1 TO
pelvic_floor_target_progress_week_12
        / pfg = pelvic_floor_goal_w1 TO pelvic_floor_goal_w12.
  COMPUTE pfg = (pfw >= 7).
END REPEAT.

DO REPEAT pfpw = pelvic_floor_physio_target_progress_week_1 TO
pelvic_floor_physio_target_progress_week_12
        / pfpg = pelvic_floor_physio_goal_w1 TO pelvic_floor_physio_goal_w12.
  COMPUTE pfpg = (pfpw >= 2).
END REPEAT.

DO REPEAT cw = cardio_target_progress_sec_week_1 TO
cardio_target_progress_sec_week_12
        / cg = cardio_goal_w1 TO cardio_goal_w12.
  COMPUTE cg = (cw >= 9000).
END REPEAT.

DO REPEAT mw = mental_target_progress_week_1 TO mental_target_progress_week_12
        / mg = mental_goal_w1 TO mental_goal_w12.
  COMPUTE mg = (mw >= 2).
END REPEAT.

DO REPEAT kw = knowledge_target_progress_week_1 TO
knowledge_target_progress_week_12
        / kg = knowledge_goal_w1 TO knowledge_goal_w12.
  COMPUTE kg = (kw >= 2).
END REPEAT.

COMPUTE weeks_goal_pelvic     = SUM(pelvic_floor_goal_w1 TO pelvic_floor_goal_w12).
COMPUTE weeks_goal_physio     = SUM(pelvic_floor_physio_goal_w1 TO
pelvic_floor_physio_goal_w12).
COMPUTE weeks_goal_cardio     = SUM(cardio_goal_w1 TO cardio_goal_w12).
COMPUTE weeks_goal_mental     = SUM(mental_goal_w1 TO mental_goal_w12).
COMPUTE weeks_goal_knowledge  = SUM(knowledge_goal_w1 TO knowledge_goal_w12).

COMPUTE total_goals_achieved  = weeks_goal_pelvic + weeks_goal_physio +
weeks_goal_cardio
                        + weeks_goal_mental + weeks_goal_knowledge.
COMPUTE percent_goals_achieved = (total_goals_achieved / 60) * 100.
VARIABLE LABELS
```

```
    weeks_goal_pelvic "Weeks pelvic-floor goal met"
    weeks_goal_physio "Weeks pelvic-floor physio goal met"
    weeks_goal_cardio "Weeks cardio goal met"
    weeks_goal_mental "Weeks mental goal met"
    weeks_goal_knowledge "Weeks knowledge goal met"
    total_goals_achieved "Total weekly goals achieved (0-60)"
    percent_goals_achieved "% of weekly goals achieved".
FORMAT percent_goals_achieved (F5.1).
EXECUTE.

MEANS TABLES=weeks_goal_pelvic weeks_goal_physio weeks_goal_cardio
           weeks_goal_mental weeks_goal_knowledge BY gruppe
  /CELLS MEAN STDDEV COUNT.


* ===============================================================.
* SECTION 7: MECHANISM - REACTIVATION.
* ===============================================================.

TITLE 'Mechanism: Reactivation Analysis'.

COMPUTE A1  = (trainings_week_1 > 0).
COMPUTE A2  = (trainings_week_2 > 0).
COMPUTE A3  = (trainings_week_3 > 0).
COMPUTE A4  = (trainings_week_4 > 0).
COMPUTE A5  = (trainings_week_5 > 0).
COMPUTE A6  = (trainings_week_6 > 0).
COMPUTE A7  = (trainings_week_7 > 0).
COMPUTE A8  = (trainings_week_8 > 0).
COMPUTE A9  = (trainings_week_9 > 0).
COMPUTE A10 = (trainings_week_10 > 0).
COMPUTE A11 = (trainings_week_11 > 0).
COMPUTE A12 = (trainings_week_12 > 0).

COMPUTE P2  = (trainings_week_1 = 0).
COMPUTE P3  = (trainings_week_2 = 0).
COMPUTE P4  = (trainings_week_3 = 0).
COMPUTE P5  = (trainings_week_4 = 0).
COMPUTE P6  = (trainings_week_5 = 0).
COMPUTE P7  = (trainings_week_6 = 0).
COMPUTE P8  = (trainings_week_7 = 0).
COMPUTE P9  = (trainings_week_8 = 0).
COMPUTE P10 = (trainings_week_9 = 0).
COMPUTE P11 = (trainings_week_10 = 0).
```

```
COMPUTE P12 = (trainings_week_11 = 0).

COMPUTE R2  = P2  AND A2.
COMPUTE R3  = P3  AND A3.
COMPUTE R4  = P4  AND A4.
COMPUTE R5  = P5  AND A5.
COMPUTE R6  = P6  AND A6.
COMPUTE R7  = P7  AND A7.
COMPUTE R8  = P8  AND A8.
COMPUTE R9  = P9  AND A9.
COMPUTE R10 = P10 AND A10.
COMPUTE R11 = P11 AND A11.
COMPUTE R12 = P12 AND A12.

COMPUTE reactivation_opportunities = SUM(P2 TO P12).
COMPUTE reactivations = SUM(R2 TO R12).
COMPUTE reactivation_rate = $SYSMIS.
IF (reactivation_opportunities > 0) reactivation_rate = reactivations / reactivation_opportunities.
VARIABLE LABELS
   reactivation_opportunities "Weeks with prior inactivity"
   reactivations "Weeks with reactivation"
   reactivation_rate "Reactivation rate".

COMPUTE any_reactivation = $SYSMIS.
IF (reactivation_opportunities > 0) any_reactivation = (reactivations > 0).
VALUE LABELS any_reactivation 0 "No" 1 "Yes".
VARIABLE LABELS any_reactivation "Any reactivation episode".
EXECUTE.

CROSSTABS /TABLES=gruppe BY any_reactivation
   /STATISTICS=CHISQ
   /CELLS=COUNT ROW COLUMN
   /MISSING=TABLE.

LOGISTIC REGRESSION VARIABLES any_reactivation
  /METHOD=ENTER age bmi is_smoker any_pharma gruppe
  /CONTRAST(gruppe)=Indicator(1)
  /CONTRAST(is_smoker)=Indicator(1)
  /CONTRAST(any_pharma)=Indicator(1)
  /PRINT=CI(95) GOODFIT
  /CRITERIA=PIN(.05) POUT(.10) ITERATE(20) CUT(.5).

MEANS TABLES=reactivation_rate BY gruppe
  /CELLS MEAN STDDEV COUNT.
```

```
GLM reactivation_rate BY gruppe
  WITH age bmi is_smoker any_pharma
  /METHOD=SSTYPE(3)
  /EMMEANS=TABLES(gruppe) WITH(age=MEAN bmi=MEAN is_smoker=MEAN
any_pharma=MEAN)
          COMPARE ADJ(BONFERRONI)
  /PRINT=ETASQ PARAMETER DESCRIPTIVE
  /CRITERIA=ALPHA(.05)
  /DESIGN=age bmi is_smoker any_pharma gruppe.

* ==================================================================.
* SECTION 8: PERSISTENCE - KAPLAN-MEIER.
* ==================================================================.

TITLE 'Persistence: Time to First Inactivity'.

COMPUTE first_inactive_week = 0.
DO IF (trainings_week_1 = 0).
  COMPUTE first_inactive_week = 1.
ELSE IF (trainings_week_2 = 0).
  COMPUTE first_inactive_week = 2.
ELSE IF (trainings_week_3 = 0).
  COMPUTE first_inactive_week = 3.
ELSE IF (trainings_week_4 = 0).
  COMPUTE first_inactive_week = 4.
ELSE IF (trainings_week_5 = 0).
  COMPUTE first_inactive_week = 5.
ELSE IF (trainings_week_6 = 0).
  COMPUTE first_inactive_week = 6.
ELSE IF (trainings_week_7 = 0).
  COMPUTE first_inactive_week = 7.
ELSE IF (trainings_week_8 = 0).
  COMPUTE first_inactive_week = 8.
ELSE IF (trainings_week_9 = 0).
  COMPUTE first_inactive_week = 9.
ELSE IF (trainings_week_10 = 0).
  COMPUTE first_inactive_week = 10.
ELSE IF (trainings_week_11 = 0).
  COMPUTE first_inactive_week = 11.
ELSE IF (trainings_week_12 = 0).
  COMPUTE first_inactive_week = 12.
ELSE.
  COMPUTE first_inactive_week = 13.
```

```
END IF.
VARIABLE LABELS first_inactive_week "Week of first inactivity (13=none)".

COMPUTE time_to_inactive = first_inactive_week.
IF (first_inactive_week = 13) time_to_inactive = 12.
COMPUTE event_inactive = (first_inactive_week < 13).
VALUE LABELS event_inactive 0 "censored" 1 "inactive".
VARIABLE LABELS
    time_to_inactive "Time to first inactive week"
    event_inactive "Event: became inactive".
EXECUTE.

KM time_to_inactive BY gruppe
  /STATUS=event_inactive(1)
  /PRINT TABLE MEAN
  /COMPARE OVERALL POOLED
  /PLOT SURVIVAL.

* ==============================================================.
* SECTION 9: CLINICAL - CGI-I.
* ==============================================================.

TITLE 'Clinical: CGI-I (Unadjusted - GLM with Bonferroni)'.

MEANS TABLES=cgi_score BY gruppe
  /CELLS MEAN STDDEV COUNT.

* Replace ONEWAY with GLM.
GLM cgi_score BY gruppe
  /METHOD=SSTYPE(3)
  /EMMEANS=TABLES(gruppe) COMPARE ADJ(BONFERRONI)
  /PRINT=DESCRIPTIVE ETASQ HOMOGENEITY
  /CRITERIA=ALPHA(.05)
  /DESIGN=gruppe.

* ==============================================================.
* SECTION 9B: ADJUSTED ANALYSIS OF CGI-I.
* ==============================================================.

TITLE 'Clinical: CGI-I (Adjusted with Covariates)'.

GLM cgi_score BY gruppe
  WITH age bmi is_smoker any_pharma
  /METHOD=SSTYPE(3)
```

```
  /EMMEANS=TABLES(gruppe) WITH(age=MEAN bmi=MEAN is_smoker=MEAN
any_pharma=MEAN)
          COMPARE ADJ(BONFERRONI)
  /PRINT=ETASQ PARAMETER DESCRIPTIVE
  /CRITERIA=ALPHA(.05)
  /DESIGN=age bmi is_smoker any_pharma gruppe.

* ===============================================================.
* SECTION 10: CLINICAL - IIEF-5.
* ===============================================================.

TITLE 'Clinical: IIEF-5 Week 12 (ANCOVA)'.

GLM iief_week_12 BY gruppe
  WITH iief_week_1 age bmi is_smoker any_pharma
  /EMMEANS=TABLES(gruppe) WITH(iief_week_1=MEAN age=MEAN bmi=MEAN
is_smoker=MEAN any_pharma=MEAN)
          COMPARE ADJ(BONFERRONI)
  /PRINT=ETASQ PARAMETER DESCRIPTIVE
  /CRITERIA=ALPHA(.05)
  /DESIGN=iief_week_1 age bmi is_smoker any_pharma gruppe.

GLM iief_week_12 BY gruppe
  WITH iief_week_1 age bmi is_smoker any_pharma
  /PRINT=PARAMETER
  /DESIGN=iief_week_1 age bmi is_smoker any_pharma gruppe
         gruppe*iief_week_1.

TITLE 'Clinical: IIEF-5 Change Score (Sensitivity - GLM with Bonferroni)'.

COMPUTE iief_change = iief_week_12 - iief_week_1.
VARIABLE LABELS iief_change "IIEF-5 change score".
EXECUTE.

MEANS TABLES=iief_change BY gruppe
  /CELLS MEAN STDDEV COUNT.

* Replace ONEWAY with GLM.
GLM iief_change BY gruppe
  /METHOD=SSTYPE(3)
  /EMMEANS=TABLES(gruppe) COMPARE ADJ(BONFERRONI)
  /PRINT=DESCRIPTIVE ETASQ HOMOGENEITY
  /CRITERIA=ALPHA(.05)
  /DESIGN=gruppe.
```

```
* ===========================================================.
* SECTION 11: IMPLEMENTATION - INTENTION TO CONTINUE.
* ===========================================================.

TITLE 'Implementation: Intention to Continue (Unadjusted)'.

CROSSTABS /TABLES=gruppe BY followon_intent_num
    /STATISTICS=CHISQ
    /CELLS=COUNT ROW COLUMN
    /MISSING=TABLE.

TITLE 'Implementation: Intention to Continue (Adjusted)'.

LOGISTIC REGRESSION VARIABLES followon_intent_num
  /METHOD=ENTER age bmi is_smoker any_pharma gruppe
  /CONTRAST(gruppe)=Indicator(1)
  /CONTRAST(is_smoker)=Indicator(1)
  /CONTRAST(any_pharma)=Indicator(1)
  /PRINT=CI(95) GOODFIT
  /CRITERIA=PIN(.05) POUT(.10) ITERATE(20) CUT(.5).

* ===========================================================.
* SECTION 11B: FIGURE 2 - BAR CHART OF CONTINUATION INTENT.
* ===========================================================.

TITLE 'FIGURE 2: Proportion Intending to Continue Therapy by Group'.

* Calculate proportions for bar chart.
AGGREGATE OUTFILE=* MODE=ADDVARIABLES OVERWRITE=YES
  /BREAK=gruppe
  /total_n=N
  /intent_yes=SUM(followon_intent_num).

COMPUTE intent_proportion = intent_yes / total_n.
VARIABLE LABELS intent_proportion "Mean intention to continue therapy".
FORMATS intent_proportion (F4.2).
EXECUTE.

* Create bar chart.
GRAPH
  /BAR(SIMPLE)=MEAN(intent_proportion) BY gruppe
  /TITLE='Proportion of Participants Intending to Continue Therapy by Group'.
```

```
* Clean up temporary variables.
DELETE VARIABLES total_n intent_yes intent_proportion.
EXECUTE.

* ===============================================================.
* SECTION 12: IMPLEMENTATION - CONVERSION.
* ===============================================================.

TITLE 'Implementation: Conversion (Unadjusted)'.

CROSSTABS /TABLES=gruppe BY followon_cvr
    /STATISTICS=CHISQ
    /CELLS=COUNT ROW COLUMN
    /MISSING=TABLE.

TITLE 'Implementation: Conversion (Adjusted)'.

LOGISTIC REGRESSION VARIABLES followon_cvr
  /METHOD=ENTER age bmi is_smoker any_pharma gruppe
  /CONTRAST(gruppe)=Indicator(1)
  /CONTRAST(is_smoker)=Indicator(1)
  /CONTRAST(any_pharma)=Indicator(1)
  /PRINT=CI(95) GOODFIT
  /CRITERIA=PIN(.05) POUT(.10) ITERATE(20) CUT(.5).

* ===============================================================.
* SECTION 13: ADHERENCE THRESHOLDS.
* ===============================================================.

TITLE 'Adherence Thresholds: ≥6, ≥8, ≥9 Weeks'.

COMPUTE adh_6plus = (Active_weeks >= 6).
COMPUTE adh_8plus = (Active_weeks >= 8).
COMPUTE adh_9plus = (Active_weeks >= 9).
VALUE LABELS
    adh_6plus 0 "No" 1 "Yes"
    / adh_8plus 0 "No" 1 "Yes"
    / adh_9plus 0 "No" 1 "Yes".
VARIABLE LABELS
    adh_6plus "Adherence: ≥6 active weeks"
    adh_8plus "Adherence: ≥8 active weeks"
    adh_9plus "Adherence: ≥9 active weeks".
EXECUTE.
```

```
CROSSTABS /TABLES=gruppe BY adh_6plus
    /STATISTICS=CHISQ
    /CELLS=COUNT ROW COLUMN.

CROSSTABS /TABLES=gruppe BY adh_8plus
    /STATISTICS=CHISQ
    /CELLS=COUNT ROW COLUMN.

CROSSTABS /TABLES=gruppe BY adh_9plus
    /STATISTICS=CHISQ
    /CELLS=COUNT ROW COLUMN.

LOGISTIC REGRESSION VARIABLES adh_8plus
  /METHOD=ENTER age bmi is_smoker any_pharma gruppe
  /CONTRAST(gruppe)=Indicator(1)
  /CONTRAST(is_smoker)=Indicator(1)
  /CONTRAST(any_pharma)=Indicator(1)
  /PRINT=CI(95) GOODFIT
  /CRITERIA=PIN(.05) POUT(.10) ITERATE(20) CUT(.5).

* ============================================================.
* SECTION 14: ALTERNATIVE ADHERENCE METRICS.
* ============================================================.

TITLE 'Alternative: Active Weeks v2 (≥2 sessions OR ≥30 min cardio)'.

COMPUTE active_week_v2_1  = (trainings_week_1 >= 2) OR
(cardio_target_progress_sec_week_1 >= 1800).
COMPUTE active_week_v2_2  = (trainings_week_2 >= 2) OR
(cardio_target_progress_sec_week_2 >= 1800).
COMPUTE active_week_v2_3  = (trainings_week_3 >= 2) OR
(cardio_target_progress_sec_week_3 >= 1800).
COMPUTE active_week_v2_4  = (trainings_week_4 >= 2) OR
(cardio_target_progress_sec_week_4 >= 1800).
COMPUTE active_week_v2_5  = (trainings_week_5 >= 2) OR
(cardio_target_progress_sec_week_5 >= 1800).
COMPUTE active_week_v2_6  = (trainings_week_6 >= 2) OR
(cardio_target_progress_sec_week_6 >= 1800).
COMPUTE active_week_v2_7  = (trainings_week_7 >= 2) OR
(cardio_target_progress_sec_week_7 >= 1800).
COMPUTE active_week_v2_8  = (trainings_week_8 >= 2) OR
(cardio_target_progress_sec_week_8 >= 1800).
COMPUTE active_week_v2_9  = (trainings_week_9 >= 2) OR
(cardio_target_progress_sec_week_9 >= 1800).
```

```
COMPUTE active_week_v2_10 = (trainings_week_10 >= 2) OR
(cardio_target_progress_sec_week_10 >= 1800).
COMPUTE active_week_v2_11 = (trainings_week_11 >= 2) OR
(cardio_target_progress_sec_week_11 >= 1800).
COMPUTE active_week_v2_12 = (trainings_week_12 >= 2) OR
(cardio_target_progress_sec_week_12 >= 1800).
COMPUTE Active_weeks_v2 = SUM(active_week_v2_1 TO active_week_v2_12).
VARIABLE LABELS Active_weeks_v2 "Active weeks v2".
EXECUTE.

MEANS TABLES=Active_weeks_v2 BY gruppe
  /CELLS MEAN STDDEV COUNT.

* Replace ONEWAY with GLM.
GLM Active_weeks_v2 BY gruppe
  /METHOD=SSTYPE(3)
  /EMMEANS=TABLES(gruppe) COMPARE ADJ(BONFERRONI)
  /PRINT=DESCRIPTIVE ETASQ HOMOGENEITY
  /CRITERIA=ALPHA(.05)
  /DESIGN=gruppe.

GLM Active_weeks_v2 BY gruppe
  WITH age bmi is_smoker any_pharma
  /METHOD=SSTYPE(3)
  /EMMEANS=TABLES(gruppe) WITH(age=MEAN bmi=MEAN is_smoker=MEAN
any_pharma=MEAN)
        COMPARE ADJ(BONFERRONI)
  /PRINT=ETASQ PARAMETER
  /CRITERIA=ALPHA(.05)
  /DESIGN=age bmi is_smoker any_pharma gruppe.

TITLE 'Alternative: Multi-Domain Weeks (≥2 of 5 domain goals)'.

COMPUTE multi_domain_w1  = ((pelvic_floor_goal_w1 + pelvic_floor_physio_goal_w1 +
cardio_goal_w1 + mental_goal_w1 + knowledge_goal_w1) >= 2).
COMPUTE multi_domain_w2  = ((pelvic_floor_goal_w2 + pelvic_floor_physio_goal_w2 +
cardio_goal_w2 + mental_goal_w2 + knowledge_goal_w2) >= 2).
COMPUTE multi_domain_w3  = ((pelvic_floor_goal_w3 + pelvic_floor_physio_goal_w3 +
cardio_goal_w3 + mental_goal_w3 + knowledge_goal_w3) >= 2).
COMPUTE multi_domain_w4  = ((pelvic_floor_goal_w4 + pelvic_floor_physio_goal_w4 +
cardio_goal_w4 + mental_goal_w4 + knowledge_goal_w4) >= 2).
COMPUTE multi_domain_w5  = ((pelvic_floor_goal_w5 + pelvic_floor_physio_goal_w5 +
cardio_goal_w5 + mental_goal_w5 + knowledge_goal_w5) >= 2).
```

```
COMPUTE multi_domain_w6  = ((pelvic_floor_goal_w6 + pelvic_floor_physio_goal_w6 +
cardio_goal_w6 + mental_goal_w6 + knowledge_goal_w6) >= 2).
COMPUTE multi_domain_w7  = ((pelvic_floor_goal_w7 + pelvic_floor_physio_goal_w7 +
cardio_goal_w7 + mental_goal_w7 + knowledge_goal_w7) >= 2).
COMPUTE multi_domain_w8  = ((pelvic_floor_goal_w8 + pelvic_floor_physio_goal_w8 +
cardio_goal_w8 + mental_goal_w8 + knowledge_goal_w8) >= 2).
COMPUTE multi_domain_w9  = ((pelvic_floor_goal_w9 + pelvic_floor_physio_goal_w9 +
cardio_goal_w9 + mental_goal_w9 + knowledge_goal_w9) >= 2).
COMPUTE multi_domain_w10 = ((pelvic_floor_goal_w10 + pelvic_floor_physio_goal_w10 +
cardio_goal_w10 + mental_goal_w10 + knowledge_goal_w10) >= 2).
COMPUTE multi_domain_w11 = ((pelvic_floor_goal_w11 + pelvic_floor_physio_goal_w11 +
cardio_goal_w11 + mental_goal_w11 + knowledge_goal_w11) >= 2).
COMPUTE multi_domain_w12 = ((pelvic_floor_goal_w12 + pelvic_floor_physio_goal_w12 +
cardio_goal_w12 + mental_goal_w12 + knowledge_goal_w12) >= 2).
COMPUTE multi_domain_weeks = SUM(multi_domain_w1 TO multi_domain_w12).
VARIABLE LABELS multi_domain_weeks "Weeks with ≥2 of 5 domain goals met".
EXECUTE.

MEANS TABLES=multi_domain_weeks BY gruppe
  /CELLS MEAN STDDEV COUNT.

* Replace ONEWAY with GLM.
GLM multi_domain_weeks BY gruppe
  /METHOD=SSTYPE(3)
  /EMMEANS=TABLES(gruppe) COMPARE ADJ(BONFERRONI)
  /PRINT=DESCRIPTIVE ETASQ HOMOGENEITY
  /CRITERIA=ALPHA(.05)
  /DESIGN=gruppe.

GLM multi_domain_weeks BY gruppe
  WITH age bmi is_smoker any_pharma
  /METHOD=SSTYPE(3)
  /EMMEANS=TABLES(gruppe) WITH(age=MEAN bmi=MEAN is_smoker=MEAN
any_pharma=MEAN)
          COMPARE ADJ(BONFERRONI)
  /PRINT=ETASQ PARAMETER
  /CRITERIA=ALPHA(.05)
  /DESIGN=age bmi is_smoker any_pharma gruppe.

* ==============================================================.
* SECTION 15: MISSING DATA & ATTRITION.
* ==============================================================.

TITLE 'Missing Data: Follow-up Completion'.
```

```
COMPUTE iief_fu = 0.
IF (NOT MISSING(iief_week_12)) iief_fu = 1.
COMPUTE cgi_fu = 0.
IF (NOT MISSING(cgi_score)) cgi_fu = 1.
VALUE LABELS
   iief_fu 0 "No" 1 "Yes"
   / cgi_fu 0 "No" 1 "Yes".
VARIABLE LABELS
   iief_fu "Completed IIEF follow-up"
   cgi_fu "Completed CGI follow-up".
EXECUTE.

CROSSTABS /TABLES=gruppe BY iief_fu
   /STATISTICS=CHISQ
   /CELLS=COUNT ROW COLUMN.

CROSSTABS /TABLES=gruppe BY cgi_fu
   /STATISTICS=CHISQ
   /CELLS=COUNT ROW COLUMN.

T-TEST GROUPS=iief_fu(0 1)
   /VARIABLES=age bmi iief_week_1 Active_weeks
   /MISSING=ANALYSIS.

LOGISTIC REGRESSION VARIABLES iief_fu
  /METHOD=ENTER age bmi is_smoker Active_weeks any_pharma gruppe
  /CONTRAST(gruppe)=Indicator(1)
  /CONTRAST(is_smoker)=Indicator(1)
  /CONTRAST(any_pharma)=Indicator(1)
  /PRINT=CI(95) GOODFIT
  /CRITERIA=PIN(.05) POUT(.10) ITERATE(20) CUT(.5).

* ================================================================.
* SECTION 16: SUBGROUP - NO BASELINE PHARMACOTHERAPY.
* ================================================================.

TITLE 'Subgroup: IIEF Outcome (No Baseline Pharmacotherapy)'.

TEMPORARY.
SELECT IF (any_pharma = 0).
GLM iief_week_12 BY gruppe
  WITH iief_week_1 age bmi is_smoker
```

```
  /EMMEANS=TABLES(gruppe) WITH(iief_week_1=MEAN age=MEAN bmi=MEAN
is_smoker=MEAN)
          COMPARE ADJ(BONFERRONI)
  /PRINT=ETASQ PARAMETER DESCRIPTIVE
  /CRITERIA=ALPHA(.05)
  /DESIGN=iief_week_1 age bmi is_smoker gruppe.

* ===================================================================.
* SECTION 17: DIAGNOSTICS.
* ===================================================================.

TITLE 'Diagnostics: Assumptions'.

EXAMINE VARIABLES=Active_weeks BY gruppe
  /PLOT=HISTOGRAM NPPLOT
  /STATISTICS=DESCRIPTIVES
  /NOTOTAL.

EXAMINE VARIABLES=iief_change BY gruppe
  /PLOT=HISTOGRAM NPPLOT
  /STATISTICS=DESCRIPTIVES
  /NOTOTAL.

GLM Active_weeks BY gruppe
  WITH age bmi is_smoker any_pharma
  /METHOD=SSTYPE(3)
  /SAVE=RESID PRED
  /PLOT=RESIDUALS
  /DESIGN=age bmi is_smoker any_pharma gruppe.

* ===================================================================.
* SECTION 17B: CORRELATIONS WITH ACTIVE WEEKS.
* ===================================================================.

TITLE 'Correlations: Active Weeks with Baseline and Clinical Variables'.

* Pearson correlation matrix including Active_weeks.
* For binary variables (is_smoker, any_pharma), Pearson r serves as point-biserial correlation.

CORRELATIONS
  /VARIABLES=Active_weeks age bmi is_smoker any_pharma iief_week_1 iief_change
  /PRINT=TWOTAIL NOSIG
  /STATISTICS=DESCRIPTIVES
  /MISSING=PAIRWISE.
```

```
* Additional correlation table focusing on Active_weeks relationships.
TITLE 'Correlations: Active Weeks - Detailed View'.

CORRELATIONS
  /VARIABLES=Active_weeks WITH age bmi is_smoker any_pharma iief_week_1 iief_change
  /PRINT=TWOTAIL SIG
  /STATISTICS=DESCRIPTIVES
  /MISSING=PAIRWISE.

* ======================================================================.
* SECTION 18: SUMMARY TABLES.
* ======================================================================.

TITLE 'Summary: All Metrics by Group'.

MEANS TABLES=Active_weeks total_trainings median_trainings_active_week
            engagement_ratio percent_goals_achieved
            Active_weeks_v2 multi_domain_weeks BY gruppe
  /CELLS MEAN STDDEV COUNT.

MEANS TABLES=iief_week_1 iief_week_12 iief_change cgi_score BY gruppe
  /CELLS MEAN STDDEV COUNT.

* ======================================================================.
* SECTION 19: FINAL VERIFICATION.
* ======================================================================.

TITLE 'Final Verification: Sample Size (N=470)'.

FREQUENCIES VARIABLES=gruppe
  /ORDER=ANALYSIS.

DESCRIPTIVES VARIABLES=Active_weeks iief_week_12 cgi_score
  /STATISTICS=MEAN STDDEV MIN MAX.

* ======================================================================.

* ======================================================================.
* SECTION 20: CALENDAR-TIME SENSITIVITY FOR SECONDARY OUTCOMES.
* ======================================================================.

TITLE 'Calendar-Time: IIEF-5 Analysis'.
```

```
REGRESSION
  /STATISTICS COEFF CI(95) R ANOVA
  /DEPENDENT iief_week_12
  /METHOD=ENTER iief_week_1 age bmi is_smoker any_pharma enrollment_time_z.

TITLE 'Calendar-Time: CGI-I Analysis'.

REGRESSION
  /STATISTICS COEFF CI(95) R ANOVA
  /DEPENDENT cgi_score
  /METHOD=ENTER age bmi is_smoker any_pharma enrollment_time_z.

* ============================================================.
* END OF SYNTAX.
* ============================================================.
```
